# Systematic Review of mHealth Interventions for Adolescent and Young Adult HIV Prevention and the Adolescent HIV Continuum of Care in Low to Middle Income Countries

**DOI:** 10.1007/s10461-022-03840-0

**Published:** 2022-11-02

**Authors:** Madeleine Goldstein, Moherndran Archary, Julian Adong, Jessica E. Haberer, Lisa M. Kuhns, Ann Kurth, Keshet Ronen, Marguerita Lightfoot, Irene Inwani, Grace John-Stewart, Robert Garofalo, Brian C. Zanoni

**Affiliations:** 1 Emory University, Atlanta, GA United States of America; 2 Children’s Healthcare of Atlanta, Atlanta, GA United States of America; 3 University of KwaZulu-Natal Nelson Mandela School of Medicine, Durban, South Africa; 4 King Edward VIII Hospital, Durban, South Africa; 5 Mbarara University of Science and Technology, Mbarara, Uganda; 6 Massachusetts General Hospital, Boston, MA United States of America; 7 Harvard Medical School, Boston, MA United States of America; 8 Feinberg School of Medicine, Northwestern University, Chicago, IL USA; 9 Ann & Robert H. Lurie Children’s Hospital of Chicago, Chicago, IL USA; 10 Yale University, Orange, CT USA; 11 University of Washington, Seattle, WA United States of America; 12 Center for AIDS Prevention Studies and UCSF Prevention Research Center, University of California San Francisco, San Francisco, CA USA; 13 Kenyatta National Hospital, Nairobi, Kenya; 14 Rollins School of Public Health, Atlanta, GA USA

**Keywords:** Adolescent, HIV, mHealth, low- and middle-income countries, implementation science

## Abstract

Adolescents and young adults (AYA) in low to middle income countries (LMIC) have poorer outcomes along each step in the HIV continuum of prevention and care compared to younger children or older adults. The use of mHealth technology provides a potentially promising implementation strategy for interventions to remedy these disparities. We therefore conducted a systematic review of the English literature and conference proceedings from January 1, 2000 to April 1, 2021 evaluating mHealth interventions targeting AYA along each step of the HIV continuum of care in LMIC. We identified 27 mHealth interventions across the HIV continuum, with no interventions addressing transition from pediatric to adult care. The majority of studies were single arm, uncontrolled or underpowered, with few randomized trials resulting in mixed and inconclusive outcomes. mHealth interventions have potential to remedy disparities along the HIV continuum of care for AYA in LMIC but larger, powered randomized trials are needed.

**Supplementary Information:**

The online version contains supplementary material available at 10.1007/s10461-022-03840-0.

## Resumen

Los adolescentes y adultos jóvenes (AYA) en países de ingresos bajos a medianos (LMIC) tienen peores resultados en cada paso del continuo de prevención y atención del VIH en comparación con los niños más pequeños o los adultos mayores. El uso de la tecnología mHealth proporciona una estrategia de implementación potencialmente prometedora para las intervenciones para remediar estas disparidades. Por lo tanto, realizamos una revisión sistemática de los resúmenes y artículos publicados en inglés desde el 1 de enero de 2000 hasta el 1 de abril de 2021 para evaluar las intervenciones de mHealth dirigidas a AYA a lo largo de cada paso del continuo de atención del VIH en LMIC. Identificamos 27 intervenciones de mHealth en todo el continuo del VIH, sin intervenciones que abordaran la transición de la atención pediátrica a la de adultos. La mayoría de los estudios fueron de un solo brazo, no controlados o con bajo poder estadístico, con pocos ensayos aleatorios que dieron resultados mixtos y no concluyentes. Las intervenciones de mHealth tienen el potencial de remediar las disparidades a lo largo de la continuidad de la atención del VIH para AYA en LMIC, pero se necesitan ensayos aleatorios más grandes y potentes.

## Introduction

In 2020, there were an estimated 1.75 million adolescents between the ages of 10 and 19 living with HIV globally, with only about half receiving antiretroviral treatment [1]. Low- to middle-income countries (LMIC) have the highest burden of adolescents and young adults (AYA) living with HIV in the world [2]. Approximately 70% of the AYA living with HIV live in LMIC [1], highlighting new and existing political, cultural, infrastructure, geographic, and economic disparities [3–5]. Adolescents experience greater vulnerability to the complexities of living with HIV given the challenges of pubertal development coupled with sexual maturation, emergence of autonomy, and social changes. Additionally, adolescents face parental consent barriers to HIV and sexual/reproductive health services, economic and power dynamic vulnerabilities, as well as insufficient access to quality and age-appropriate sexual education. AYA living with perinatally-acquired HIV, in particular, experience different psychosocial, behavioral, and medical challenges compared to AYA living with behaviorally-acquired HIV, including higher rates of mental illness and potential for substance abuse, delayed pubertal development, and increased potential for emergence of antiretroviral drug resistance [6–8]. In addition to increasing numbers of AYA with perinatally-acquired HIV surviving into adulthood, approximately 31% of new HIV infections worldwide in 2020 were among adolescents aged 15–24 most commonly through sexual transmission, with approximately three-quarters of these occurring in adolescent girls [1]. Although there is growing recognition of the need for improved adolescent HIV prevention and care, HIV/AIDS remains a leading cause of death among all adolescents in LMIC [9].

AYA in particular experience poorer HIV outcomes at each step of the care continuum compared to younger children or older adults. Despite the scale up of global antiretroviral therapy (ART), including international guidelines supporting early initiation of ART with simple, potent, and well-tolerated regimens [10], adolescents have lower rates of HIV diagnosis, engagement in care, and viral suppression compared to adults [11–14], resulting in approximately only 35% of estimated total number of adolescents who are living with HIV in LMIC being virally suppressed [1]. In addition, many adolescents require an additional step in the continuum of care during healthcare transition from pediatric to adult services contributing additional challenges to engagement in care, adherence to medications, and viral suppression after transition [15–17].

mHealth, the use of mobile wireless technologies for health, may improve the disparities along the AYA HIV continuum of care. Greater than 80% of the population in LMIC access health information and services through mHealth that can address healthcare delivery gaps across the care continuum [18–23]. mHealth is convenient, highly scalable, and has the potential to engage AYA who have high utilization rates of technology and mobile phone ownership worldwide [24, 25]. Additionally, mHealth interventions from short message service (SMS) to complex “smartphone” applications have been shown to impact several factors that improve AYAs’ engagement in care along the continuum, including relationships, social support, healthcare access, and knowledge [26–30]. Although results have varied in different settings, a recent meta-analysis showed overall improved adherence and viral suppression among adults living with HIV using mHealth technology [21]. Despite the increase in mHealth interventions in LMIC, there is a paucity of studies evaluating the implementation of these interventions for the prevention and treatment of HIV among AYA.

Our systematic review evaluates mHealth interventions addressing the HIV continuum of care, including HIV prevention, HIV testing, linkage to care, adherence/retention in care, viral suppression and transition to adult care that have been designed for and evaluated among AYA in LMIC.

## Methods

We searched PubMed and Google Scholar from January 1, 2000, to April 1, 2021 and online conference proceedings from the International AIDS Society (IAS), International Workshop on Pediatric HIV/AIDS, the International AIDS Conference (AIDS), the Conference on Retrovirology and Opportunistic Infections (CROI), and HIV Research 4 Prevention (R4P) from January 1, 2019, to April 1, 2021. Key words and medical subject headings relevant to age (i.e., adolescent, adolescence, teen, youth, young adults) were cross-referenced with terms associated with mHealth (mHealth, digital, app, web, internet, SMS/Short Messaging Service, text message, WhatsApp, Facebook, phone, mobile, device, social media, geosocial, telegram, TikTok, mxit) and each step in the HIV continuum of care (i.e., prevention, PrEP, diagnosis, linkage to care, retention in care, adherence, and transition). See **Appendix 1** for search terms. We conducted six independent searches for each step in the HIV continuum of care. To be eligible, studies had to meet the following inclusion criteria: (1) original peer-reviewed studies published in English; (2) involve mHealth interventions addressing HIV prevention and the AYA HIV continuum of care that have been piloted, implemented, or evaluated in LMIC (as defined by the World Bank Group); and (3) include interventions where the median age of participants was between 10 and 25 years. For inclusion in our review, we defined interventions as having been purposefully designed and prospectively assigned to influence bio/behavioral outcomes related to HIV. We defined mHealth as clinical and public health practices supported by the provision of health services and information via mobile technologies, such as mobile phones, tablet computers and personal digital assistants (PDA) [31]. We excluded reviews and protocol papers. We reported age ranges as described by the original authors. We adhered to the Preferred Reporting Items for Systematic Reviews and Meta-Analyses (PRISMA) guidelines.

Following the search, all identified references were imported into Covidence, a systematic review management system, and were de-duplicated. Eight authors (MG, BZ, JH, JA, LK, KR, II, and GJS) screened the remaining citations by title, abstract, and full-text against pre-determined, aforementioned inclusion and exclusion criteria by independent reviewers for each step in the HIV continuum of care, with discrepancies resolved by consensus.

### Data Analysis

After meeting our inclusion and exclusion criteria, we then extracted data from the full text articles/abstracts and created summarized tables organized by each step in the HIV continuum of care. We created a template for study extraction including study characteristics and findings. The template included the following: authors, title, year, country, age range, number of subjects, intervention description, and results of the intervention. For studies that included samples from multiple age groups, we extracted only the data that pertained to adolescents and young adults.

## Results

**Fig. 1:**
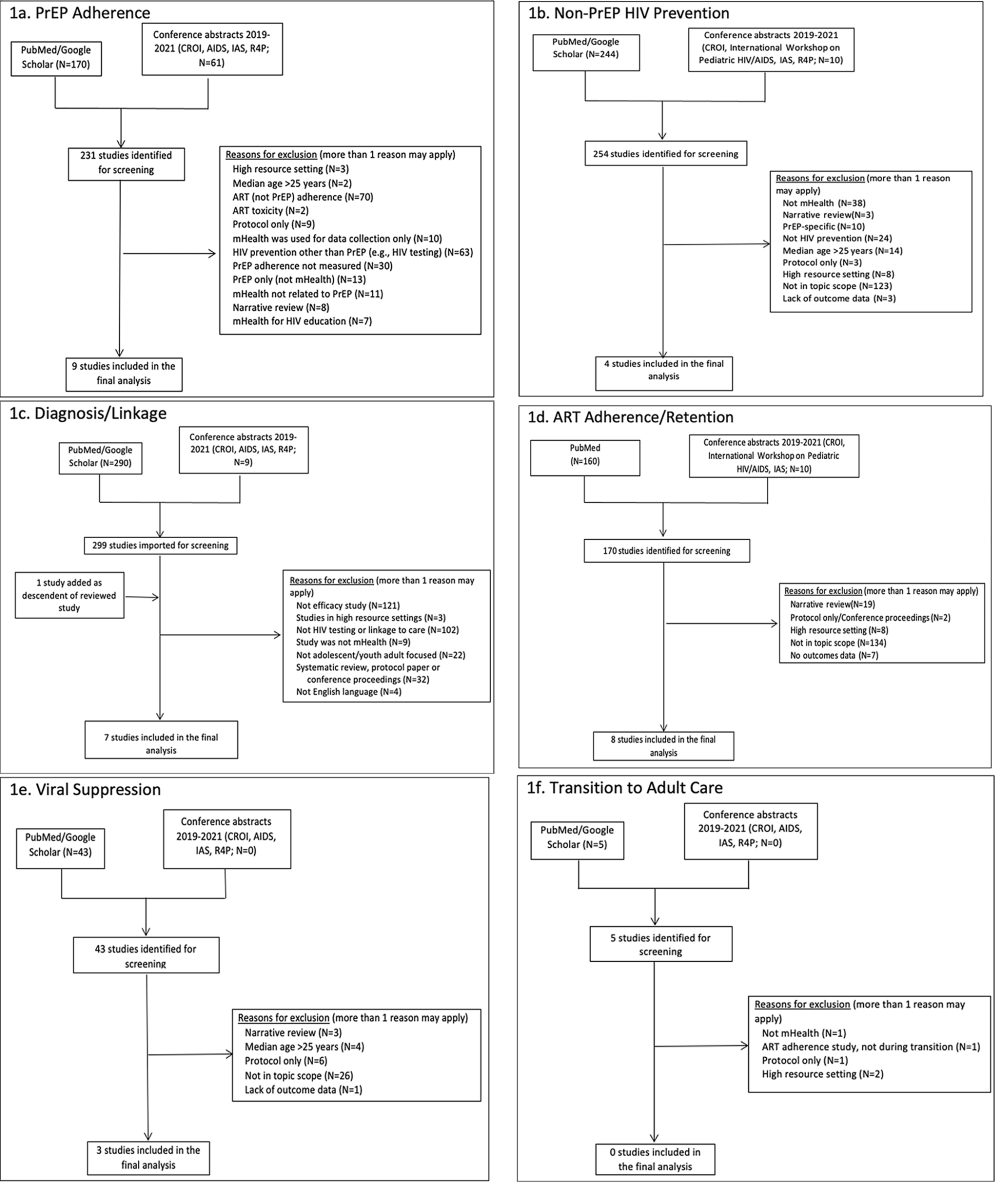
Results of search and inclusion/exclusion

**Fig. 2:**
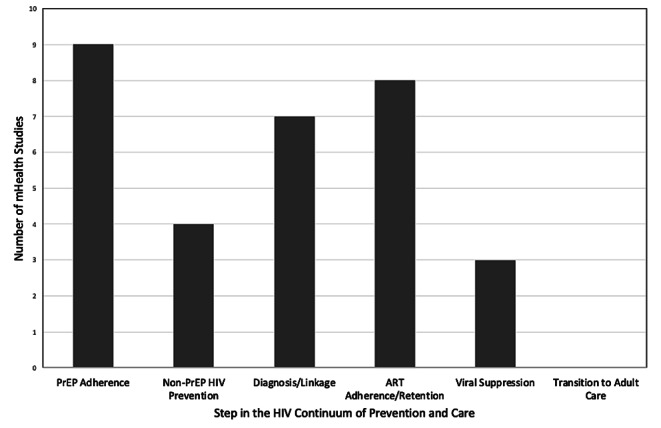
mHealth studies addressing each step in the HIV continuum of prevention and care

Overall, we reviewed 912 articles and 90 conference abstracts in our initial screening (Fig. 1). We identified 27 studies where mHealth was used as or with an intervention addressing the AYA HIV continuum of care in LMIC with several interventions meeting criteria for more than one step in the continuum (Table 1; **Fig. **2). We found thirteen studies addressing HIV prevention including nine pre-exposure prophylaxis (PrEP) related and four non-PrEP prevention interventions. Seven studies addressed HIV testing and linkage to care with one study overlapping with prevention and one with retention in care. Eight interventions addressed ART adherence and engagement in care. An additional three studies addressed viral suppression with two overlapping with adherence and retention in care. There were no mHealth interventions addressing transition from pediatric to adult based care in LMIC. Additionally, nineteen of the studies measured implementation outcomes including feasibility, acceptability, and/or uptake of the intervention in addition to HIV clinical outcomes. We found that five interventions utilized social media [32–36]; fifteen used SMS- or telephone-based messaging including education, counseling, reminders, and peer mentoring (37–39, 40–41, 42, 43, 44–45, 46, 47–36); and seven interventions included smartphone- or tablet-based applications [51–57].

**Table 1:** mHealth Interventions to improve the HIV continuum of care for AYA in LMIC

Authors	Title	Publication and date	Location	Number of subjects	Ages	Study type	Intervention description	Results of intervention/discussion
**PrEP Adherence**								
Beyrer C, et al. [32]	Social media influencers enhance recruitment of young Thai MSM into PrEP intervention	CROI, 2020	Thailand	Not stated	Not stated	Observational	• Compared PrEP intervention enrolment without and with social media influencers	• Rate of recruitment increased from 17 to 63.3/month (82% increase [95% CI = 31-154%; p-value < 0.001]); 75% choose PrEP initiation • Study demonstates the use of social media influencers to increase the engagement and enrolment of at-risk individuals from traditional health structures
Celum CL, et al. [37]	PrEP adherence and effect of drug level feedback among young African women in HPTN 082	IAS, 2019	Cape Town and Johannesburg, South Africa; Harare, Zimbabwe	427	21	2-arm RCT	• Standard adherence support (counselling, 2-way SMS, and adherence clubs) vs. standard support + drug level feedback • Adherence assessed by TFV-DP (DBS) and TFV levels (plasma)	• No difference by arms in proportion of TFV-DP or high adherence by DBS or plasma TFV (all p > 0.3) • Addition of drug level feedback to standard adherence support did not improve PrEP adherence
Celum CL, et al. [38]	Incentives conditioned on tenofovir levels to support PrEP adherence among young South African women: a randomized trial (the 3Ps for Prevention study)	Journal of the International AIDS Society, 2020	Cape Town, South Africa	200	19 (17–21)	2-arm RCT	• Standard of care vs. conditioned incentive (conditioned on level of plasma TDF) • Follow up at Month 4 by phone (the only mHealth component of the intervention) • Primary outcome was PrEP adherence defined as TFV-DP ≥700 fmol/punch at month 3	• No sustained difference in PrEP adherence between the groups, 56% intervention group vs. 41% control group had TDF-DP ≥700 fmol/punch at Month 3; RR = 1.35 (0.98–1.86, p = 0.067) • Over half persisted with 12-month PrEP program • High adherence observed at Month 3, but declined thereafter
Celum CL, et al. [39]	PrEP initiation, persistence, and HIV seroconversion rates in African adolescent girls and young women from Kenya and South Africa: The POWER demonstration project	R4P, 2021	Cape Town and Johannesburg, South Africa; Kisumu, Kenya	2469	21 (19–23)	Observational	• Multiple implementation models (mobile truck, adolescent-friendly clinic, publc clinics, family planning clinic) plus SMS/WhatsApp/phone call for PrEP refill reminders	• 2315 (94%) initiated PrEP, 688 (30%) of whom returned at 1 month for a refill; of those who returned at 1 month, 21% persisted with PrEP for 6 months and 9% for 12 months • Of 1972 who returned after a refill gap of > 1 month, 509 (26%) restarted PrEP
Dourado I, et al. [33]	Go seek: reaching youth and adolescents’ men who have sex with men and trangender woman to offer PrEP in Brazil	AIDS, 2020	Brazil	446	Median age not stated; (15–19)	Observational	• Peer and social media-based interventions to recruit MSM and TGW with a goal of PrEP uptake	• Peer-based PrEP recruitment yielded more PrEP initiators compared to social media-based recruitment strategies
Haberer J, et al. [40]	Effects of SMS reminders on PrEP adherence in young Kenyan women (MPYA study): a randomised control trial	Lancet HIV, 2021	Thika and Kisumu, Kenya	348	21 (19–22)	2-arm RCT	• 1-way SMS reminders vs. no reminders • Reminders were initially sent daily and participants could switch to as-needed reminders after 1 month • Evaluated PrEP adherence over 24 months	• No difference between groups; adherence averaged 27% with SMS vs. 26.6% without SMS; adjusted incidence rate ratio 1.16 (0.93–1.45, p = 0.19) • Additional approaches are needed to support PrEP adherence
Pintye J, et al. [41]	Two-Way Short Message Service (SMS) Communication May Increase Pre-Exposure Prophylaxis Continuation and Adherence Among Pregnant and Postpartum Women in Kenya	Global Health Science and Practice, 2020	Kusumu, Kenya	334	25 (22–30)	Prospective cohort study	• Adapted an existing 2-way SMS platform to send PrEP-tailored, theory-based SMS and allow clients to communicate with a remote nurse • Evaluated PrEP continuation at 1-month post initiation of intervention compared to women who initiated PrEP 1 month before intervention implementation	• Intervention group were more likely to return at their one month follow-up visit (40% vs. 53%; adjusted risk ratio = 1.26 [1.06–1.50, p = 0.008]) • Intervention group had better PrEP retention (22% vs. 43%; aRR = 1.75 (1.21–12.55, p = 0.003)) • Intervention group had better comprehension of PrEP
Songtaweesin WN, et al. [51]	Youth-friendly services and a mobile phone application to promote adherence to pre‐exposure prophylaxis among adolescent men who have sex with men and transgender women at‐risk for HIV in Thailand: a randomized control trial	Journal of the International AIDS Society, 2020	Bangkok, Thailand	200	18 (17–19)	2-arm RCT	• Prospective RCT randomized 1:1 to receive Youth friendly services (YFS) vs. YFS plus use of a PrEP adherence mobile phone app (YFS + APP) • Features of app included: Self assessment of HIV acquisition risk, point rewards, adherence and clinic reminders. • Primary outcome was PrEP adherence defined as TFV-DP ≥700 fmol/punch	• No difference between groups in PrEP adherence (Month 3: 51% YFS vs. 54% YFS + APP, p = 0.64; at month 6: 44% YFS vs. 49% YFS + APP, p = 0.54) • YFS PrEP services enabled good adherence among half of users; there was no additional benefit from the PrEP app
Songtaweesin WN, et al. [42]	High PrEP adherence based on TFV-DP levels in Thai 15-19-year-old MSM and transgender women	CROI, 2020	Thailand	148	Median age not stated; (15–19)	Observational	• MSM and TGW provided with PrEP and condoms in a youth-friendly clinic and contacted monthly by phone • Risk of HIV acquisition was assessed by TFV-DP levels and condom use	• Youth friendly clinic and monthly follow-ups provided a 72% HIV risk reduction among MSM and TGW • Monthly phone calls, in the context of other supportive services, may contribute to PrEP adherence
**Non-PrEP HIV Prevention**								
Garg PR, et a^§^ [52]	Mobile Health App for Self-Learning on HIV Prevention Knowledge and Services Among a Young Indonesian Key Population: Cohort Study	JMIR Mhealth Uhealth, 2020	Indonesia (Jakarata, West Java, East Java, Special Region of Yogyakarta, and Bali)	168	MSM 22.6; Transgender women 25.6; PWUD 23.7 (16–30)	Prospective intervention cohort study	• A peer-customized mobile app (RumahSELA) based on the principle of self learning in increasing knowledge on HIV transmission, creating awareness about safe sex and HIV prevention, facilitating behavioral change, and fostering the uptake of HIV testing for improving access to health services • Pre-post assessment	• There was a significant increase in comprehensive HIV-related knowledge among all 3 groups (p = 0.004 MSM, p < 0.001 transgender women, p < 0.001 PWUD) • 34% of the participants took an HIV test after using the app; with an uptake in HIV testing by 31% for MSM, 49% for transgender women, and 26% for PWUD after using the app. • A reduction was observed in the number of individuals who did not use condoms in their last sexual intercourse encounter postintervention in all three groups (MSM (P = 0.45), transgender women (P = 0.25), and PWUD (P < 0.001)). • The app was highly accepted by the key populations and more than half of participants found the app to be user friendly.
Visser M, et a^§^ [53]	An mHealth HIV prevention programme for youth: lessons learned from the iloveLife.mobi programme in South Africa	AIDS Care, 2020	South Africa	• KAPB survey (n = 1,882) • Group discussions (n = 68) • Interviews with users (n = 175) and project and community stakeholders (n = 46)	24.2 (13–45)	Mixed-methods (qualitative and retrospective cohort)	• Behavioral change intervention that uses mHealth to promote positive health behavior through access to information such as sexual and reproductive health and psychosocial information on self-esteem, relationships, peer pressure, substance abuse, career opportunities and access to youth-friendly health services • Intervention mobile site uses interactive learning through short articles, audio drama, quizzes, self-assessments and discussion forums • Conducted retrospective surveys, FG discussions, and interviews with users and project/community stakeholders • High-frequency users were considered those who used site every day or a few times a week	• High-frequency users were more likely to report that the site motivated them to protect themselves against HIV, use condoms consistently, know their HIV status, use contraceptives, undergo VMMC and to be more positive about their future (p < 0.001) vs. low-frequency users. • High-frequency users were more confident that they will be able to abstain from sex, insist on condom use and taking an HIV test (p < 0.001) vs. low-frequency users
Winskelll K, et al. [54]	A Smartphone Game-Based Intervention (Tumaini)^a^ to Prevent HIV Among Young Africans: Pilot Randomized Controlled Trial	JMIR, 2018	Kisumu, Kenya	60	12.7 (11–14)	2-arm RCT	• Interactive, narrative-based game designed to help prevent HIV by delaying first sex and increasing condom use at first sex • Intervention group used the app for ≥1 h/day for 16 days versus control group • Primary outcome measured included behavioral mediators of sexual debut and condom use • Intervention group participants and their parents participated in focus group discussions on game feedback	• Intervention arm showed significant gains in sexual health-related knowledge and self-efficacy (p < 0.001), behavioral intention for risk-avoidance strategies and sexual risk communication (p = 0.006) at 6 weeks post-intervention compared to control group • Focus groups identified a wide range of knowledge and skills participants had gained, including setting goals and planning how to achieve them.
Ybarra M, et al. [43]	Iterative Development of In This toGether, the First mHealth HIV Prevention Program for Older Adolescents in Uganda	AIDS and Behavior, 2020	Uganda	34	19.7 (18–22)	Beta testing with 2-arm RCT	• Text messaging-based HIV prevention program (5–11 messages per day over 2 months) for both sexually-active and abstinent 18–22 year olds • Attention-matched control group that received content about healthy lifestyle topics (e.g., diet and exercise) • Participants completed brief text message-based surveys evaluating the program experience	• Intervention is both feasible and acceptable to 18–22 year olds across sexual experience levels. • Higher rates of HIV testing in Intervention group (93%) vs. control group (77%) (p = 0.24). All participants reported negative HIV tests. • Similar numbers in the intervention (50%) and control (40%) groups reported having at least one condomless sex act since the beginning of the study (p = 0.76).
**Diagnosis/Linkage**								
Adeagbo O, et al. [55]	Acceptability of a tablet-based application to support early HIV testing among men in rural KwaZulu-Natal, South Africa: a mixed method study	AIDS Care, 2021	Rural KwaZulu-Natal, South Africa	232 (subsample of a larger trial enrolled in in-depth interviews)	22.5 (18–40)	Single arm, post-only pilot trial	• Tablet-based, self-administered intervention tailored to men, EPIC-HIV to increase internal motivation to test for HIV and link to care in rural setting, based on self-determination theory • Facilitated by fieldworkers at the point of rapid HIV testing in home-based setting, as part of annual population-based surveillance • Designed to be administered in 5–10 min	• 83% (192/232) uptake of HIV testing • Intervention was acceptable among participants, especially 15–24 years old
Das A, et al. [34]	Getting to the First 90: Incentivized Peer Mobilizers Promote HIV Testing Services to Men Who Have Sex With Men Using Social Media in Mumbai, India	Glob Health: Sci Prac, 2019	Mumbai, India	247	69% less than 25	Single arm, post-only trial	• Project Mulakat included geosocial and social media outreach by MSM peer internet outreach workers to promote HIV testing at a collaborating key population clinic. Tested individuals were provided with coupons for network-based HIV testing to extend the reach of the intervention	• 247 MSM tested • Seroprevalence 3.2% (8/247) • 244 (99%) first-time testers • 50% (4/8) linked to care
Garg PR, et a^§^ [52]	Mobile Health App for Self-Learning on HIV Prevention Knowledge and Services Among a Young Indonesian Key Population: Cohort Study	JMIR MHealth Uheatlh, 2020	Jakarta, West Java, East Java, Special Region of Yogyakarta, and Bali provinces of Indonesia	168	MSM 22.6; Transgender women 25.6; PWUD 23.7 (16–30)	Prospective intervention cohort study	• Android-based mobile app, RumahSELA, with risk assessment, “ask a question” to a heath provider, schedule an HIV test, map of health facilities, educational games, quizzes, prizes. • Youth leaders administered the app	• Self-reported uptake of HIV testing from baseline to 3-months increased from 79% (133/168) to 90% (151/168), a change of 11% (p < 0.035) • PWUD group, 65% (46/70) to 82% (57/70) change of 17% (*P* < 0.001), • MSM from 88–94% and transgender women 96–98%. • 34% reported an HIV test after using the app, 21% (35/168) made the appointment through the app. • 24/49, 49% transgender women, 31% (15/49) and 26% (18/70) of the MSM and PWUD reported an HIV test after using the app. • 27% (7/24) MSM, 25% (4/15) transgender women, and 11% (2/18) PWUD had made the appointment through the app.
Hacking D, et al.^§^ [44]	Peer Mentorship via Mobile Phones for Newly Diagnosed HIV-Positive Youths in Clinic Care in Khayelitsha, South Africa: Mixed Methods Study	JMIR, 2019	Urban informal settlement of Khayelitsha, South Africa	105 (35 intervention, 70 matched control)	12–25	Prospective matched case-control study	• Each mentee matched to 2 control patients with similar HIV testing and counselling dates • Medecins Sans Frontieres stand-alone, youth-only clinic with youth-specific services, including mobile-phone support groups via WhatsApp, and “virtual mentor” program linking newly diagnosed youth to peer mentors (HIV positive youth) via mobile-phone • 2 to 8 weeks of mentoring provided, depending on their recruitment date • Focused on patients who declined to join a youth adherence club	• 28 (80%) linked to ART in intervention vs. 30 (43%) control. • Days to linkage was 217.5 (32.5-467.75) in the intervention group; 49.5 (7-333.25) in the matched control
Njuguna N, et al. [45]	The Effect of Human Immunodeficiency Virus Prevention and Reproductive Health Text Messages on Human Immunodeficiency Virus Testing Among Young Women in Rural Kenya: A Pilot Study	Sex Transm Dis, 2016	Kiambu County, Central Kenya	600	21 (18–24)	Quasi-experimental study with randomization of 4 sites (2 intervention, 2 control)	• Sought to increase HIV testing through increases in knowledge, increasing risk perception and decreasing risk behavior via SMS text messages. • Weekly SMS messages on HIV and reproductive health-related topics • Six categories of topics (63 messages): pregnancy, contraceptives, STIs, condoms, anal and oral sex, HIV risk • Could request additional messages on same topic, up to 3 additional messages per week.	• 59% (356/600) reported testing, 67% (201/300) in the intervention group and 51% (155/300) in the control group (log rank, p < 0.0001) • 57% (95% CI: 28-92%) difference in the intervention group • Median time to first test was 12 weeks versus 20 weeks in the intervention and control groups respectively
Pant Pai N, et al. [56]	Will an innovative connected AideSmart! app-based multiplex, point-of-care screening strategy for HIV and related coinfections affect timely quality antenatal screening of rural Indian women? Results from a cross-sectional study in India	Sex Trans Infect, 2019	Vellore District, India	510	24 years (17–40)	Single arm, retrospective comparison	• Integrated, digital, point-of-care testing (POCT) strategy for HIV (and other conditions), AideSmart!, via finger prick rapid test. • Healthcare professionals (HCP) screen pregnant women with POCTs in rural service units near their homes and navigate through the AideSmart! System. • Secure tablet application with web link to care and digital data collection • Each HCP followed 35 women	• In the 6 months prior to intervention, 296/510 screened for HIV; at intervention 100% (510/510) screened for HIV; an increase of 42% • Seroprevalence 0%
Visser M, et al.^§^ [53]	An mHealth HIV prevention programme for youth: lessons learned from the iloveLife.mobi programme in South Africa	AIDS Care, 2020	South Africa	1882 (survey respondents)	24.2 (13–45)	Mixed-methods (qualitative and retrospective cohort)	• iloveLife.mobi web app, to reduce risk behavior, promote uptake of HIV testing and voluntary medical male circumcision • Target youth ages 12–24 • Site uses interactive learning through games, quizzes, self-assessments, and discussion forums • Points given for engagement on the site and participation in events in-person, includes leader board. • Site promoted through social media and wider promotion (radio)	• In a retrospective survey, 87% of respondents reported having tested for HIV (83% of young men and 89.5% of young women) during the program period
**ART Adherence/Retention**								
Abiodun O, et al.^§^ [46]	A Single-Blind, Parallel Design RCT to Assess the Effectiveness of SMS Reminders in Improving ART Adherence Among Adolescents Living with HIV (STARTA Trial)	J Adolesc Health, 2021	Southwest Nigeria	209	16.61 (15–19)	2-arm RCT	• Daily ART reminder SMS • Clinic visit reminder SMS 24 and 48 h prior to appointment • Participants asked to respond to each SMS with “1” to indicate message was acceptable and “2” otherwise • Intervention duration 20 weeks • All participants had < 95% self-reported adherence at enrollment	• No significant difference between arms in self-reported ART adherence. OR of VAS report ≥ 95% in intervention vs. control 1.20 (0.91–1.58, p = 0.25) • VL 0.66 log_10_ copies/ml (95% CI 0.26–1.06, 0 = 0.001) lower in intervention vs. control; OR of VL ≤ 20c/ml in intervention vs. control 1.36 (1.04–1.77, p = 0.002) • 83.4% of SMS successfully delivered; 26.2% required multiple attempts • 62.5% of SMS responded to; 95.3% responded that SMS was acceptable
Dulli L, et al. [35]	A Social Media–Based Support Group for Youth Living With HIV in Nigeria (SMART Connections): Randomized Controlled Trial	J Med Internet Res, 2020	South-central Nigeria	353	21.3 (15–24)	2-arm RCT	• Virtual facilitated group of 15–25 YLWH, using Facebook secret group • In-person launch meeting then ~ daily activities, including social activities, interactive polls, facilitated discussions, educational images & written messages • Intervention duration 22 weeks • All participants received a smartphone and 1GB data bundle per month • All participants had been on ART for < 12 months	• No significant difference between arms in retention in care at 270 days. Probability of retention in control arm 0.45 (0.36–0.54) vs. intervention arm 0.50 (0.41–0.58) • No significant difference between arms in self-reported ART adherence (p = 0.57) • Significant increase in HIV-related knowledge in intervention group (p = 0.003)
Hacking D, et al.^§^ [44]	Peer Mentorship via Mobile Phones for Newly Diagnosed HIV-Positive Youths in Clinic Care in Khayelitsha, South Africa: Mixed Methods Study	J Med Internet Res, 2019	Cape Town, South Africa	125	Median age not stated; (12–25)	Matched case-control	• Peer-to-peer phone-based pairing of newly diagnosed YLWH with trained YLWH stable in care • Mentor sent 2 greeting messages by SMS, WhatsApp or call and invite mentee to attend youth adherence club. Additional unscripted interaction encouraged • Intervention duration 2–8 weeks	• Increased ART initiation (80% vs. 43%) and first VL test completion (80% vs. 45%) in intervention group • No difference in retention in care at 6 months (92% in intervention vs. 89% in control) and 12 months (100% vs. 94%) after ART initiation • No difference in VL suppression (< 400 c/ml) at 4 months after ART initiation in intervention (93%) vs. control (91%)
Ivanova O, et al. [57]	Evaluation of the ELIMIKA Pilot Project: Improving ART Adherence among HIV Positive Youth Using an eHealth Intervention in Mombasa, Kenya	Afr J Reprod Health, 2019	Mombasa, Kenya	90	18.4 (15–24)	Prospective cohort	• Interactive web-based peer support platform for YLWH including a blog written by project coordinators, Q&A with providers, stories contest, private messaging. • Intervention duration 3 months	• No significant difference in ART adherence post- vs. pre-intervention (71.6% missed no doses at enrollment vs. 77.8% at follow-up, p = 0.95)
Linnemayr S, et al. [47]	Text Messaging for Improving Antiretroviral Therapy Adherence: No Effects After 1 Year in a Randomized Controlled Trial Among Adolescents and Young Adults	Am J Public Health, 2017	Kampala, Uganda	332	18 (15–22)	3-arm RCT	• 1-way weekly SMS checking participant’s status; 2-way weekly SMS checking participant’s status and requesting a reply “1” if well and “2” if unwell. Unwell responses received a phone call within 24 h • Intervention duration 48 weeks • All participants were provided with a MEMS cap for adherence measurement	• No significant difference in mean adherence over intervention period between arms: control 67%, 1-way 64% (p-value vs. control = 0.27), 2-way 61% (p-value vs. control = 0.15)
MacCarthy S, et al. [48]	A randomized controlled trial study of the acceptability, feasibility, and preliminary impact of SITA (SMS as an Incentive To Adhere): a mobile technology-based intervention informed by behavioral economics to improve ART adherence among youth in Uganda	BMC Infect Dis, 2020	Kampala, Uganda	155	Median age not stated; (15–24)	3-arm pilot RCT	• T1 intervention: weekly 1-way SMS informing them of their Wisepill adherence level in previous week; T2 intervention: weekly information about their Wisepill adherence and peer Wisepill adherence • Intervention duration 36 weeks • All participants had ≥ 20% adherence by Wisepill in a 2-month observation pre-randomization	• No significant difference in Wisepill ART adherence. Adherence in T1 vs. control − 3.8% (-9.9-2.3%) adherence; T2 vs. control + 2.4% (-3.0-7.9%) adherence • Intervention was deemed feasible and acceptable
Ronen K, et al. [49]	Improved ART knowledge and adherence skills in youth living with HIV participating in a WhatsApp support group in Nairobi, Kenya: The Vijana-SMART pilot study	IAS 2020	Nairobi, Kenya	55	18 (14–24)	Prospective cohort	• Facilitated WhatsApp group with ~ 25 YLWH. Weekly facilitator messages and group discussion • Intervention duration 6 months	• No significant difference in ART adherence pre- vs. post-intervention. 88.9% (77.8–94.4%) adherence at enrollment vs. 92.2% (77.8–97.8%) at follow-up, p = 0.50 • Increase in ART information and behavioral skills pre- vs. post-intervention (p < 0.001)
Sánchez SA, et al. [50]	Toward improved adherence: a text message intervention in an human immunodeficiency virus pediatric clinic in Guatemala City	Medicine, 2021	Guatemala City, Guatemala	143	Median age not stated; (6–24)	2-arm RCT	• One-way SMS 3 times per week • Messages addressed taking ART consistently, taking ART at the same time every day, instructions on how to take ART • Participants age 6–12: SMS addressed to caregiver; age 13–17: addressed to participant or caregiver per participant choice; age ≥ 18: addressed to participant • Intervention duration 6 months • Patients with VL > 400c/ml or living at orphanages at baseline were excluded	• 4% increase in mean adherence score from enrollment to 6-month follow-up in intervention arm (p < 0.01). 0.85% increase in control (p = 0.64) • Consistent results stratified by age group • 69.9% of participants completed follow-up. Challenges with phone number changes and retaining contact noted
**Viral Suppression**								
Abiodan O, et al.^§^ [46]	A Single-Blind, Parallel Design RCT to Assess the Effectiveness of SMS Reminders in Improving ART Adherence Among Adolescents Living with HIV (STARTA Trial).	J Adol Health, 2021	Nigeria	212 randomized, 209 analyzed medication non-adherent adolescents	Median age not stated; (15–19)	Parallel RCT	• Interactive tailored SMS on ART adherence among adolescents • SMS at 48 and 24 h before visit • ART adherence reminders daily • Participants required to reply SMS as soon as possible – 1 if they found reminder acceptable depending on the circumstance around them, 2 if it was not so acceptable.	• Log_10_ VL difference between arms at 20 week follow-up 0.66 (95% CI 0.26–1.06, p = 0.001). • Undetected viral load ≤ 20 copies/ml OR 1.356 (95% CI 1.039–1.771, p = 0.002).
Hacking D, et al.^§^ [44]	Peer Mentorship via Mobile Phones for Newly Diagnosed HIV-Positive Youths in Clinic Care in Khayelitsha, South Africa: Mixed Methods Study.	J Med Internet Res, 2019	South Africa	35 intervention; 70 matched controls	Median age not stated; (12–25)	Mixed methods	• Virtual mentor via WhatsApp or SMS • Peer-to-peer phone-based pairing of newly diagnosed YLWH with trained YLWH stable in care • Mentor sent 2 greeting messages by SMS, WhatsApp or call and invite mentee to attend youth adherence club. Additional unscripted interaction encouraged • Intervention duration 2–8 weeks	• Linkage to ART and VL testing were significantly higher in virtual mentor (VM) group than controls (80 vs. 43% for linkage and 80 vs. 45% for VL testing in VM vs. control, respectively). • Viral suppression was similar between virtual mentor arm and controls (93 vs. 91%, VM vs. control).
Stankievich E, et al. [36]	Utility of Mobile Communication Devices as a Tool to Improve Adherence to Antiretroviral Treatment in HIV-infected Children and Young Adults in Argentina.	Ped Infect Dis, 2018	Argentina	25 virally non-suppressed individuals	< 25	Pre-post	• Text messages, WhatsApp, or facebook • Youth asked their preferred method • Generic mobile message sent twice a month • Messages short questions about status of patient and medication-related issues • If patient or parent wanted further information a feedback phone call or contact by message could be generated • Required reply to verify receipt • 32 week duration	• All unsuppressed to start, following intervention 65% (13/20) undetectable, 70% (14/20) < 1000 c/ml and 6 with no change in VL
**Transition to Adult Care**								
No studies fit search criteria								

**Footnotes**:

^**a**^Three additional publications examining the acceptability, feasibility, and mechanisms of effect of Tumaini [58–60]

^§^This study addresses more than one step in the HIV continuum of care

### HIV Prevention

We identified seventeen studies addressing HIV prevention, including pre-exposure prophylaxis (PrEP) related and non-PrEP prevention interventions. Independent searches for PrEP related and non-PrEP HIV prevention interventions were conducted to individually highlight the intervention strategies and outcomes for both the biomedical and behavioral approaches to HIV prevention.

### PrEP Adherence

We identified nine studies in which mHealth was used as or with an intervention to promote PrEP adherence among AYA in LMIC (Fig. 1a). These studies were conducted in sub-Saharan Africa, Brazil, and Thailand. Our search was based on a broad interpretation of adherence, including initiation, execution, and persistence of PrEP use [61]. The majority of studies focused on older adolescents and young adults [33, 37–42, 51]. The technology approaches to PrEP adherence included smartphone-based psychoeducational and medication adherence apps; SMS or telephone-based educational and adherence support messages; and social media outreach. These approaches were often facilitated by peers (e.g., youth, key populations) or healthcare workers (e.g., nurses).

Four randomized controlled trials (RCTs) have been published to date, none of which found a benefit from mHealth [38, 40, 51]. Haberer et al. used one-way SMS reminders among 348 young women in Kenya who were considered at high risk of HIV acquisition. Adherence was assessed by electronic monitoring and pharmacy refill over 24 months; neither differed between the intervention and control arms [40]. Songtaweesin et al. developed an app that included self-assessment of HIV acquisition risk, point rewards, and reminders for medication adherence and clinic attendance [51]. This intervention was studied in combination with youth-friendly services versus youth-friendly services alone among 200 men who have sex with men (MSM) and transgender women (TGW) at-risk for HIV in Thailand; no difference was seen in tenofovir diphosphate (TFV-DP) levels between the two study arms. In the 3Ps for Prevention study, Celum et al. randomized 200 young women in South Africa to receive or not receive financial incentives conditioned of TFV-DP levels; mHealth was utilized for follow-up involving a phone call at Month 4 [38]. Again, no difference in executed adherence was seen in the intervention and control arms. Additionally, in HPTN 082, Celum et al. compared standard adherence support (counselling, 2-way SMS, and adherence clubs) with standard support plus drug level feedback in an RCT among young women in South Africa and Zimbabwe [37]. No difference was seen in the two arms.

Our search of abstracts revealed five additional studies. In a conference abstract about the POWER demonstration project, Celum et al. reported high PrEP initiation but low persistence when PrEP was delivered in multiple youth-friendly models in South Africa and Kenya; SMS, WhatsApp, and phone calls were utilized for PrEP refill reminders [39]. Pintye et al. conducted a pilot prospective cohort study among 334 pregnant and post-partum women in Kenya in which a two-way SMS platform was used to promote communication with the clinic [41]. Compared to a pre-intervention group, women starting PrEP were more likely to return to clinic and to continue taking PrEP at one month; longer follow-up was not presented. In a conference abstract, Dourado et al. compared peer and social media-based interventions to recruit 446 MSM and TGW in Brazil for PrEP initiation; peer recruitment was found to be more effective [33]. Beyrer et al. presented a conference abstract in which they showed that social media influencers enhanced recruitment of young Thai MSM into a PrEP intervention with 75% PrEP initiation through short scenario-based videos with health messaging that reached a large audience [32]. Finally, in another conference abstract, Songtaweesin et al. used monthly phone calls in combination with a youth-friendly clinic to promote PrEP adherence and condom use among 148 MSM and TGW in Thailand [42]. This approach was found to be effective with a 72% HIV risk reduction, although the effectiveness of the mHealth piece alone was not clear given the observational nature of the study and the combination intervention.

### Non-PrEP HIV Prevention

Our search found four interventions that have been published addressing mHealth interventions for non-PrEP related HIV prevention among AYA in LMICs (Fig. 1b). These studies were conducted in both urban and rural areas of: Indonesia, South Africa, Uganda, and Kenya. One study focused on older adolescents and young adults ages 18–22 [43], one included younger adolescents 11–14 years [54], and two focused on broader age ranges [52, 53]. Two RCTs have been published to date; however, both were underpowered for clinical outcomes [43, 54]. In the remaining HIV prevention studies, one was a prospective cohort [52] and the other was a retrospective cohort study [53]. Interventions for non-PrEP HIV Prevention included smartphone applications with psychoeducational content, interactive learning, and narrative-based games, as well as SMS-driven prevention messages. The content for these interventions were often developed with the assistance of peers (e.g., youth, key populations) and incorporated peer interaction within the intervention.

Several studies were published on the development, pilot testing and outcomes of Tumaini, an interactive narrative-based game aimed to prevent HIV through improving health-related knowledge and skills. The preliminary studies examining the acceptability, feasibility, and mechanisms of effect of the mHealth intervention [58–60] were excluded from the primary analysis for lack of outcome data. The primary outcome data described by Winskell et al. included a 2-arm RCT among 60 adolescents in Kenya [54]. Compared to the control group, adolescents gained sexual health-related knowledge, self-efficacy, behavioral intention for risk-avoidance strategies, and sexual risk communication skills at 6 weeks post-intervention; longer follow up and prevention rates of HIV-acquisition were not presented.

The three remaining HIV prevention studies targeted increased HIV prevention knowledge and decreasing HIV risk behaviors. Ybarra et al. conducted a beta test of In This toGether, a text-messaging based HIV prevention program aimed to increase condom use and STI/HIV testing, among 34 older adolescents in Uganda [43]. They found similar rates of condomless sexual encounters between intervention and control groups. Visser et al. conducted a mixed methods retrospective cohort evaluation of ilovelove.mobi, a mobile site that uses interactive learning through short articles, audio drama, quizzes, self-assessments, and discussion forums, to evaluate behavioral change for HIV prevention in South Africa [53]. Through retrospective surveys, the study found that youth ages 15–24 were more likely to report consistent condom use, obtain HIV testing, and undergo voluntary medical male circumcision after using the mHealth intervention compared to a comparable national sample, however surveys relied on self-report and the study did not have baseline or control groups. An additional three-arm prospective cohort study of RumahSELA, a peer-customized mobile app that aims to improve HIV prevention knowledge and access to health services, was conducted in Indonesia among 200 adolescents and young adults including MSM, TGW, and people who use drugs (PWUD) [52]. Pre-post survey assessments showed statistically significant improvements in comprehensive HIV-related knowledge from 20 to 60% among MSM, 22–57% among TGW, and 49–74% among PWUD. The study also found a reduction in the number of individuals who did not use condoms in their last sexual encounter postintervention among all three groups.

### Diagnosis/Linkage

We identified seven mHealth interventions addressing HIV diagnosis and linkage to care among AYA in LMIC (Fig. 1c). These studies took place in both urban and rural areas of four different LMICs: India, Indonesia, Kenya, and South Africa. Six of the seven interventions reported HIV testing outcomes [34, 45, 52, 53, 55, 56], while two reported linkage to HIV care outcomes [34, 44] Interventions were focused on reaching general populations of young men and women [44, 45, 53, 55], including two studies that included younger adolescents [44, 53], two that focused on older adolescents and young adults [45, 52], and four that included broader age ranges [34, 53, 55, 56]. Interventions included pregnant women, [56] as well as key populations, including MSM, transgender individuals and PWUD [34, 52] who demonstrate disproportionately low levels of HIV testing or linkage to care. The evaluation designs were dominated by single-arm post-test only or retrospective approaches [34, 52, 55, 56], with one prospective case-control study [44] and one cluster randomized trial [45].

The technology approaches to promote HIV testing and linkage to care included tablet-based education and service linkage facilitated by field workers; smartphone and web-based psychoeducational and HIV testing facilitation apps; SMS-based educational messages; and social media and geosocial outreach. These approaches were very often facilitated by peers (e.g., youth, key populations, HIV-positive youth).

In terms of the tablet-based, facilitated approaches, an interactive tablet-based psychosocial app tailored to men, EPIC-HIV, was used by fieldworkers in rural KwaZulu-Natal, South Africa, to promote uptake of rapid HIV testing and linkage to care by targeting intrinsic motivation and self-determination. The fieldworkers facilitated the intervention immediately prior to an invitation to complete testing as part of an annual HIV surveillance program [55]. Similarly, in rural Vellore District of India, healthcare navigators engaged pregnant women with tablet-based app, AideSmart!, with education to promote point-of-care (POC) screening for HIV, among other conditions [56]. Testing data were also collected via the app to communicate POC test results directly with healthcare facilities for linkage to care. Both interventions were tested in single arm designs and demonstrated high post-intervention HIV testing uptake: 83% and 100% respectively, however, only the second study had a pre-test comparison, which was for the 6 months prior to the intervention (i.e., 58% at pretest and 100% at posttest, an increase of 42% points).

Two interventions used existing social media apps to promote HIV testing and linkage to care among youth. In Mumbai, India, Project *Mulakat* sought to increase HIV testing among MSM via peer mobilization in virtual spaces, including social and geosocial media (e.g., PlanetRomeo, Facebook, Grindr) [34]. Initial “seeds” were recruited to promote HIV testing through a coupon-based system to networks of peers on social media platforms. Those interested in HIV testing were directed to test at a local collaborating clinic. Similarly, Hacking et al. describe a study conducted in the urban informal settlement of Khayelitsha, South Africa, HIV-positive mentors supported newly diagnosed peers for 2 to 8 weeks, to fully link to care via mobile phone messaging and support groups using WhatsApp, known as the “virtual mentors” program. New patients, ages 12–25, who were not yet on ART or who had declined to join the youth adherence club were eligible for the intervention [44]. Project *Mulakat* was evaluated in a single arm post-test only design, finding relatively low seroprevalence of 3.2%, with a high percentage of first-time testers (99%), but only 50% linked to care. The virtual mentors program used a prospective case-control design, with each mentee matched to two control patients with similar HIV testing and counseling dates. In the intervention group 80% linked to ART versus 43% in the control group, although the time to linkage was much longer in the intervention group.

Two interventions used smartphone or web apps to promote HIV testing. As described above, Garg and colleagues developed an Android-based app, RumahSELA, to promote HIV testing among key populations including MSM, TGW and PWUD in several provinces of Indonesia, facilitated by peers [52]. In addition to sexual education, including games and quizzes and prizes, a risk assessment, an “ask a question” feature, managed by health care providers in collaborating clinics, and a map of area health facilities, participants were able to schedule an HIV test through the app. Also as described above, Visser and colleagues developed the iloveLife.mobi website to promote HIV prevention and testing among youth aged 12–24 in South Africa [53]. Activities were incentivized with a point system and leader board and weekly “lucky draws” for prizes. In a prospective intervention cohort study, RumahSELA participants significantly increased uptake of HIV testing from 79 to 90% pre-post, with the largest increase among PWUD. However, the percent of tests linked to use of the app was relatively low overall, with the most tests among TGW. The iloveLife.mobi app was evaluated in a post-hoc retrospective survey with 87% of respondents reporting HIV testing during the program period.

Finally, in Kambu County, Central Kenya, an intervention targeted to young college women promoted HIV testing via SMS text messages focused on sexual and reproductive health by increasing knowledge, risk perception and promoting risk reduction [45]. Weekly messages were sent with the option to receive up to 3 additional messages per week upon request. The intervention was tested in a cluster randomized trial (4 sites; 2 technical colleges and 2 training colleges), finding that 67% of young women tested in the intervention sites versus 51% in the control sites: a significant difference of 57% with the time to test also occurring more rapidly in the intervention group.

### ART Adherence/Retention

Our search yielded seven peer-reviewed articles and one conference abstract reporting effect of mHealth interventions on ART adherence or retention in care in LMIC youth (Fig. 1d). Of the 8 studies identified, all but one were conducted in sub-Saharan Africa; the other study was conducted in Guatemala (Table 1). Five of the studies focused on older adolescents and young adults (ages 14–25), one focused on older adolescents age 15–19 only and two focused on broader age ranges. Four studies were powered randomized controlled trials [35, 46, 47, 50] and one was a pilot trial underpowered for clinical outcomes [48]. One study was a case-control analysis [44] and two were pre-post comparisons in observational cohorts [57]. Study sample sizes ranged from 55 to 353 participants. One study evaluated retention in care [44], one study evaluated both retention in care and ART adherence [35], and the remaining 6 studies evaluated only ART adherence. One study utilized the Theoretical Framework of Acceptability to measure acceptability outcomes [48].

Four studies reported SMS-based interventions [46–48, 50], three reported other internet-based messaging applications such as WhatsApp or Facebook [35, 49, 57] and one study used a variety of phone communication methods depending on participant preference. Two studies used digital medication dispensers in addition to messaging [47, 48], including one intervention that provided participants with data on their own monitored medication adherence. Five interventions used one-to-one communication between the participant and the study or a peer mentor, while three additionally facilitated group interactions among participants [35, 49, 57]. Three interventions provided one-way communication, meaning participants received messages but were not able to engage in dialog [46, 48, 50]; the other five interventions were interactive, facilitating either bidirectional messaging or a phone call from the study if the participant requested help. Intervention duration ranged from 2 to 48 weeks.

Of the 8 studies included, one reported a significant association between the intervention and ART adherence and one reported significant improvement in viral suppression [46, 50]. Sánchez et al. reported that in a RCT among 143 Guatemalan participants age 6–24 with suppressed VL at enrollment, those receiving one-way SMS 3 times per week exhibited a 4% increase in mean self-reported ART adherence score from enrollment to 6 months, compared with a 0.85% increase in the control arm [50]. Abiodun et al.’s RCT among 209 adolescents age 15–19 in Nigeria reported 36% increased odds of undetectable viral load after 20 weeks of daily ART reminder SMS and visit reminders (24 and 48 h prior), but no increase in self-reported ART adherence [46]. The three other RCTs and three observational studies found no significant difference in ART adherence or retention in HIV care between participants who did and did not receive mHealth interventions. However, Ronen et al. and Dulli et al. reported increased HIV-related or ART adherence knowledge in youth receiving peer group social media interventions [35, 49], and Hacking et al. (as described above in diagnosis/linkage) reported increased ART initiation and initial VL test completion in youth receiving peer-to-peer phone mentoring [44].

### Viral Suppression

Our search identified three peer-reviewed articles evaluating mHealth interventions on viral load or viral suppression among AYA in LMICs (Fig. 1e) [36, 44, 46]. Two of the studies evaluating viral suppression overlapped with adherence/retention in care [44, 46] and were conducted in sub-Saharan Africa. The third study was conducted in Argentina.

The three studies which met inclusion criteria used varied platforms (WhatsApp, Facebook, or SMS), all included the option for SMS messaging. The mHealth interventions were delivered by peers (youth living with HIV) in one study, while the others included SMS-based messages from research staff and healthcare providers. Two studies focused on non-adherent/non-suppressed youth and one on youth newly initiating ART; only one study was an RCT. Abiodun et al.’s RCT (as described above in adherence/retention) evaluated an SMS intervention among youth in Nigeria who were non-suppressed and found a significantly higher rate of viral suppression at 20 weeks follow-up in the intervention arm [46]. The two remaining studies included a mixed methods study and a pre-post study. Stakievich et al.’s pre-post study focused on children and youth who were not virally suppressed and utilized youths’ platform of choice to engage twice monthly on adherence with the option of escalating to further interaction [36]. This study found that the majority of unsuppressed children/youth became suppressed (70% <1000 c/ml and 65% undetectable) during 32 weeks of follow-up but did not include formal statistical testing of post- vs. pre-viral suppression [36]. Hacking et al.’s mixed methods study (as described above in testing and linkage) utilized virtual mentors who worked with AYA newly initiating ART and found significant improvement in linkage to care and VL testing, however, the study was underpowered to detect effects on viral suppression [44].

### Transition to Adult Care

Our systematic review found did not find any relevant articles in the published literature of mHealth interventions addressing healthcare transition for AYA living with HIV in LMICs as indicated in **Fig. **1 f.

## Discussion

AYA comprise a growing number of individuals living with HIV in LMIC; however, the percentage of AYA who are aware of their diagnosis, retained in care and virally suppressed remains low [1, 11–14]. The increased use of mHealth allows for a wide variety of novel intervention content and delivery targeting large populations along the HIV continuum of prevention and care at a potentially lower cost than human resource-intensive interventions. For youth in particular, studies involving mHealth interventions show high rates of acceptability [48, 55, 58, 62]. Our review of the literature identified several types of mHealth interventions that engage youth living in LMIC along the HIV continuum of care, including prevention, diagnosis/linkage to care, adherence/retention, and viral suppression. These interventions included simulation video games, smartphone app-delivered health information, SMS-delivered messages, social media forums, and interactive web-based peer support.

Many of the studies used mHealth as an implementation strategy as a component of a more complex intervention; however, few studies used implementation science frameworks or measured implementation outcomes such as cost, fidelity, and/or sustainability, thus, limiting the ability to measure the effectiveness of mHealth in isolation. In studies that did not find benefit when using mHealth, it is unclear whether the intervention itself, the implementation strategy, study design or lack of power, contributed to ineffectiveness. Future studies involving mHealth should be conceptualized within an implementation science framework to adequately measure effectiveness of mHealth as an implementation strategy.

Despite the popularity of mobile communications among youth generally and mHealth within the HIV field, a relatively small number of mHealth interventions have been developed to promote PrEP adherence among adolescents in resource-limited settings [33, 37–42, 51]. Among the rigorous RCTs identified in the literature, SMS reminders and a multi-feature PrEP app were not effective in promoting adherence [40, 51]. Given these studies did not measure implementation outcomes including costing, sustainability, or scale, our understanding of the intervention implementation process and effectiveness of the mHealth component of these interventions is limited. Two other RCTs included elements of mHealth (i.e., follow-up by phone and 2-way SMS adherence support), although these studies primarily assessed the impact of conditional incentives and drug-level feedback; neither approach improved PrEP adherence [37, 38]. Given that elements of mHealth were utilized as an implementation strategy of these broader interventions, it is difficult to measure their individual effectiveness. Using an implementation science framework in these complex interventions has the potential to provide a foundation from which we can measure mHealth effectiveness in addition to PrEP adherence outcomes. Two other studies that were designed to assess enhanced strategies for communication with the clinic (a two-way SMS platform and monthly phone calls) were more encouraging in promoting both execution and persistence, although they were observational and follow-up was limited [41, 42]. An implementation study also used SMS, WhatsApp, and phone calls as reminders for PrEP refills, but persistence was low and the impact of this communication is unknown [39]. Additionally, the ability of social media-based recruitment for PrEP initiation was variable [32, 33]. The literature on mHealth interventions for PrEP adherence in the United States is more promising. A recent narrative review identified three published mHealth interventions that improved execution and/or persistence among a variety of youth and adult populations: a multi-component intervention, a two-way communication platform, and an educational/skill building game progress [63]. At least eleven other mHealth studies addressing PrEP adherence are in progress.

Case finding and diagnosis is the critical first step in the HIV care continuum and AYA in LMIC are at high risk of going undiagnosed [64]. We identified seven mHealth interventions with varied technology approaches in LMIC to promote HIV diagnosis and linkage to care, the majority of which focused on HIV testing rather than linkage to care. These included tablet-based education and service linkage facilitated by field workers; smartphone and web-based psychoeducational and HIV testing facilitation apps; SMS-based educational messages; and social media and geosocial outreach. Four of the interventions were designed and targeted specifically to AYA [44, 45, 52, 53] and three had primary peer-based components [34, 44, 52], approaches which are considered best practices for reaching AYA at risk of or living with HIV [65]. Five of the six interventions demonstrated significantly higher rates of HIV testing in the intervention group versus the control group or pre-post in single arm studies, or a high rate of testing posttest-only [45, 52, 53, 55, 56]; while one (of two) demonstrated a higher rate of linkage to care at follow up in the intervention condition versus control [44]. Evaluation designs were dominated by single-arm approaches and lack measured implementation outcomes overall, which weakens findings. However, even with this weakness, the results largely support initial efficacy of mHealth approaches to promote HIV testing and linkage to care among populations of AYA in LMIC, particularly key populations. Despite high incidence and lower case finding among AYA, targeted and systematic efforts to reach them are not yet widespread making mHealth interventions an attractive potential solution.

There was a shortage of adequately powered rigorously designed evaluations of mHealth interventions for ART adherence and retention in care in YLWH. We found only four randomized evaluations that were conducted with the primary aim of assessing clinical effect [35, 46, 47, 50]. mHealth was used to deliver text message-based reminders and adherence information in the majority of these RCTs. These studies’ sample sizes were modest (143–353). Similarly, there were scant and mixed data on impact of mHealth interventions on viral suppression. The remaining studies’ non-randomized or pilot designs provide less definitive evidence resulting in mixed findings in which SMS reminders and communication platforms have been beneficial for some, but not all populations [35, 44, 46–50, 57]. Additionally, while several of these studies measured feasibility and/or acceptability of the intervention, it is difficult to assess what components of the interventions were most impactful.

There is a lack of mHealth interventions targeting AYA living with HIV transitioning to adult-oriented care despite an expected oncoming wave of youth living with HIV aging into adulthood and requiring adult-oriented services in the next 5–10 years [66–72]. Despite studies showing poor clinical outcomes associated with healthcare transition, there is a paucity of interventions targeting this population. While our review showed that there are currently no evidence-based mHealth interventions focused on healthcare transition among youth living with HIV in LMIC, there was one protocol paper detailing a smart-phone app, iTransition, which is a Social Cognitive Theory-based mobile health intervention being developed for AYA living with HIV transitioning to adult care in the United States [73]. Additionally, a social media based mHealth intervention for AYA living with undergoing healthcare transition in South Africa, InTSHA, is currently being pilot tested [74]. The development and implementation of mHealth interventions are needed to improve healthcare transition outcomes for youth living with HIV in LMIC.

Despite the overall increase in mobile phone ownership and use in recent years worldwide, AYA remain a challenging population to effectively engage in mHealth, especially in LMIC. In particular, mHealth interventions have difficulty engaging individuals of lower socioeconomic status, especially AYA who may not have their own phone or the ability to pay for data plans. Previous studies have shown that while most AYA in LMICs have access to a mobile phone, it can be either through owning or through borrowing one from someone else, including parents, siblings, friends, or other family members [75]. Several of the included interventions noted similar implementation limitations, including Sànchez et al. who noted operational challenges such as multiple users of single cell phones and a fluctuation of cell phone possession [50]. However, even if AYA have access to a cell phone, simple text message interventions alone may not be enough to capture the attention of recipients [47]. While AYA are enthusiastic to use new innovative mobile technologies to address barriers along the HIV continuum of care, some of these interventions may require increased costs for large data plans for mobile applications versus SMS-driven interventions. Additionally, poor technological literacy and inferior network coverage compared to high income countries may pose as a barrier to mHealth uptake in LMIC [75, 76]. mHealth technology has the ability to overcome AYA’s fears of stigma, discrimination, or lack of privacy when seeking HIV prevention or care by using a more private and convenient methodology compared to in-person services. However, there are certain sociocultural beliefs and expectations surrounding fear of disclosure, especially given the common practice of borrowing phones, that may limit mHealth use in LMICs.

While there is increasing use of mHealth interventions to improve health outcomes along the HIV continuum of prevention and care for AYA in LMIC, study outcomes are mixed. This is in part due to intervention design and difficulty evaluating individual intervention components, in addition to a lack of large-scale randomized-controlled trials powered to detect group differences. Although several studies comment on acceptability and/or feasibility of the intervention, there is a lack of rigorous implementation design in order to assess key components of the intervention and its implementation process. Future studies can utilize an implementation framework including using mHealth as an implementation strategy in combination with other interventions (e.g., peer support, self-efficacy) to measure intervention effectiveness, costing, fidelity and sustainability.

This review had several limitations. We included mHealth interventions that were specifically targeting AYA in LMIC and did not include larger studies with broader age ranges where some youth were included. Additionally, mHealth is a broad term and comprises a variety of interventions of varying intensities, from simple text messages to more complex interventions with interactive games and peer interaction, making an evaluation of mHealth interventions challenging. Many studies described in this review were one-off or short term-interventions. Studies of long-term engagement in care, adherence, and viral suppression are required. Many of the mHealth intervention were smaller pilot studies that have not been scaled widely, despite their promise. Despite these weaknesses, the results from most studies largely support the potential efficacy of mHealth approaches among populations of youth in LMICs, particularly key populations.

## Conclusion

The use of mHealth technology is a promising implementation strategy to address the disparities along the HIV continuum of prevention and care for AYA in LMIC. There is a high degree of acceptability and feasibility of mHealth among AYA; however, results of studies are conflicting due to predominance of uncontrolled-single arm studies with only a few adequately powered randomized trials. There is a need to utilize the principles of implementation science to better design, evaluate, and eventually scale, delivery of effective mHealth interventions for AYA in LMIC. While none of the studies evaluated mHealth interventions during the COVID-19 pandemic, the ability of mHealth to be delivered without face-to-face contact is an attractive service delivery model during a pandemic and should be further evaluated.

## Electronic Supplementary Material

Below is the link to the electronic supplementary material.

Supplementary Material 1
