# Supplementary material for: Systematic Review of mHealth Interventions for Adolescent and Young Adult HIV Prevention and the Adolescent HIV Continuum of Care in Low to Middle Income Countries
Source: AIDS Behav. 2022 Nov 2;27(Suppl 1):94–115. doi: 10.1007/s10461-022-03840-0 (PMC9629200; doi:10.1007/s10461-022-03840-0)
Supplement: Supplementary file 1 — Supplementary Material 1 [file 10461_2022_3840_MOESM1_ESM.docx]

**Appendix 1: Search Terms**

| PrEP Adherence | ((adolescent[MeSH Terms]) OR (young adult[MeSH Terms]) OR (teen*) OR (youth*) OR (adoles*) OR (young adult*)) AND ((cell phone[MeSH Terms]) OR (smartphone[MeSH Terms]) OR (computer, handheld[MeSH Terms]) OR (wireless technology[MeSH Terms]) OR (internet[MeSH Terms]) OR (mhealth[TIAB]) OR (social media[MeSH Terms]) OR (mobile applications[MeSH Terms]) OR (app[TIAB]) OR (web[TIAB]) OR (digital*[TIAB]) OR (internet[TIAB]) OR ("sms"[TIAB]) OR (whatsapp[TIAB]) OR (facebook[TIAB]) OR (grindr[TIAB]) OR (telegram[TIAB]) OR (tiktok[TIAB]) OR (geosocial[TIAB]) OR (mxit[TIAB])) AND ((Afghanistan[TIAB]) OR ("Burkina Faso"[TIAB]) OR (Burundi[TIAB]) OR ("Central African Republic"[TIAB]) OR (Chad[TIAB]) OR (congo[TIAB]) OR (Eritrea[TIAB]) OR (Ethiopia[TIAB]) OR (gambia[TIAB]) OR (guinea[TIAB]) OR ("Guinea-Bissau"[TIAB]) OR (Haiti[TIAB]) OR ("Democratic People's Republic of Korea"[Mesh]) OR (korea[TIAB]) OR (liberia[TIAB]) OR (madagascar[TIAB]) OR (malawi[TIAB]) OR (mali[TIAB]) OR (mozambique[TIAB]) OR (niger[TIAB]) OR (rwanda[TIAB]) OR ("sierra leone"[TIAB]) OR ("south sudan"[TIAB]) OR (sudan[TIAB]) OR ("syrian arab republic"[TIAB]) OR (syria[TIAB]) OR (tajikistan[TIAB]) OR (togo[TIAB]) OR (uganda[TIAB]) OR (yemen[TIAB]) OR (angola[TIAB]) OR (algeria[TIAB]) OR (bangladesh[TIAB]) OR (benin[TIAB]) OR (bhutan[TIAB]) OR (bolivia[TIAB]) OR ("cabo verde"[TIAB]) OR (cambodia[TIAB]) OR (cameroon[TIAB]) OR (comoros[TIAB]) OR ("cote d'ivoire"[TIAB]) OR ("Côte d'Ivoire"[TIAB]) OR (Djibouti[TIAB]) OR (Egypt[TIAB]) OR ("El Salvador"[TIAB]) OR (Eswatini[TIAB]) OR (Ghana[TIAB]) OR (Honduras[TIAB]) OR (India[TIAB]) OR (kenya[TIAB]) OR (Kiribati[TIAB]) OR ("Kyrgyz Republic"[TIAB]) OR ("Lao PDR"[TIAB]) OR (Laos[TIAB]) OR (Lesotho[TIAB]) OR (Mauritania[TIAB]) OR (Micronesia[TIAB]) OR (Moldova[TIAB]) OR (Mongolia[TIAB]) OR (Morocco[TIAB]) OR (Myanmar[TIAB]) OR (Nepal[TIAB]) OR (Nicaragua[TIAB]) OR (Nigeria[TIAB]) OR (Pakistan[TIAB]) OR ("Papua New Guinea"[TIAB]) OR (Philippines[TIAB]) OR ("São Tomé and Principe"[TIAB]) OR (Senegal[TIAB]) OR ("Solomon Islands"[TIAB]) OR (SriLanka[TIAB]) OR ("Sri Lanka"[TIAB]) OR (Tanzania[TIAB]) OR ("Timor-Leste"[TIAB]) OR (Tunisia[TIAB]) OR (Ukraine[TIAB]) OR (Uzbekistan[TIAB]) OR (Vanuatu[TIAB]) OR (Vietnam[TIAB]) OR ("West Bank"[TIAB]) OR (Gaza[TIAB]) OR (Zambia[TIAB]) OR (Zimbabwe[TIAB]) OR (Albania[TIAB]) OR ("American Samoa"[TIAB]) OR (Argentina[TIAB]) OR (Armenia[TIAB]) OR (Azerbaijan[TIAB]) OR (Belarus[TIAB]) OR (Belize[TIAB]) OR (Bosnia[TIAB]) OR (Herzegovina[TIAB]) OR (Botswana[TIAB]) OR (Brazil[TIAB]) OR (Bulgaria[TIAB]) OR (China[TIAB]) OR (Colombia[TIAB]) OR ("Costa Rica"[TIAB]) OR (Cuba[TIAB]) OR (dominica[TIAB]) OR ("dominican republic"[TIAB]) OR ("equatorial guinea"[TIAB]) OR (ecuador[TIAB]) OR (fiji[TIAB]) OR (gabon[TIAB]) OR (georgia[TIAB]) OR (grenada[TIAB]) OR (guatemala[TIAB]) OR (guyana[TIAB]) OR (indonesia[TIAB]) OR (iran[TIAB]) OR (iraq[TIAB]) OR (jamaica[TIAB]) OR (jordan[TIAB]) OR (Kazakhstan[TIAB]) OR (kosovo[TIAB]) OR (lebanon[TIAB]) OR (libya[TIAB]) OR (malaysia[TIAB]) OR (maldives[TIAB]) OR ("marshall islands"[TIAB]) OR (mexico[TIAB]) OR (montenegro[TIAB]) OR (namibia[TIAB]) OR ("north macedonia"[TIAB]) OR (paraguay[TIAB]) OR (peru[TIAB]) OR ("russian federation"[TIAB]) OR (russia[TIAB]) OR (samoa[TIAB]) OR (serbia[TIAB]) OR ("south africa"[TIAB]) OR ("st. lucia"[TIAB]) OR ("st. vincent"[TIAB]) OR (grenadines[TIAB]) OR (suriname[TIAB]) OR (thailand[TIAB]) OR (tonga[TIAB]) OR (turkey[TIAB]) OR (Turkmenistan[TIAB]) OR (tuvalu[TIAB]) OR (venezuela[TIAB]) OR (asia[TIAB]) OR (pacific[TIAB]) OR ("latin america"[TIAB]) OR (Caribbean[TIAB]) OR ("middle east"[TIAB]) OR (Africa[TIAB]) OR ("North africa"[TIAB]) OR ("east asia"[TIAB]) OR ("south asia"[TIAB]) OR ("sub-Saharan Africa"[TIAB]) OR ("east africa"[TIAB]) OR ("west africa"[TIAB]) OR ("north africa"[TIAB]) OR ("low-income"[TIAB]) OR ("lower-middle-income"[TIAB]) OR ("upper-middle-income"[TIAB]) OR ("low middle-income"[TIAB])) AND ((HIV infection[MeSH Terms]) OR (acquired immune deficiency syndrome[MeSH Terms]) OR (HIV[TIAB]) OR ("AIDS"[TIAB]) OR ("acquired immune deficiency syndrome"[TIAB]) OR ("acquired immunodeficiency syndrome"[TIAB])) AND (2000/1/1:2021/4/1[pdat]) AND ((adheren*[TIAB]) OR (retention[TIAB]) OR ("Pre-exposure prophylaxis"[TIAB]) OR ("Treatment Adherence and Compliance"[Mesh]) OR ("PrEP"[Mesh])) |
| --- | --- |
| Non-PrEP HIV Prevention | ((adolescent[MeSH Terms]) OR (young adult[MeSH Terms]) OR (teen*) OR (youth*) OR (adoles*) OR (young adult*)) AND ((cell phone[MeSH Terms]) OR (smartphone[MeSH Terms]) OR (computer, handheld[MeSH Terms]) OR (wireless technology[MeSH Terms]) OR (internet[MeSH Terms]) OR (mhealth[TIAB]) OR (social media[MeSH Terms]) OR (mobile applications[MeSH Terms]) OR (app[TIAB]) OR (web[TIAB]) OR (digital*[TIAB]) OR (internet[TIAB]) OR ("sms"[TIAB]) OR (whatsapp[TIAB]) OR (facebook[TIAB]) OR (grindr[TIAB]) OR (telegram[TIAB]) OR (tiktok[TIAB]) OR (geosocial[TIAB]) OR (mxit[TIAB])) AND ((Afghanistan[TIAB]) OR ("Burkina Faso"[TIAB]) OR (Burundi[TIAB]) OR ("Central African Republic"[TIAB]) OR (Chad[TIAB]) OR (congo[TIAB]) OR (Eritrea[TIAB]) OR (Ethiopia[TIAB]) OR (gambia[TIAB]) OR (guinea[TIAB]) OR ("Guinea-Bissau"[TIAB]) OR (Haiti[TIAB]) OR ("Democratic People's Republic of Korea"[Mesh]) OR (korea[TIAB]) OR (liberia[TIAB]) OR (madagascar[TIAB]) OR (malawi[TIAB]) OR (mali[TIAB]) OR (mozambique[TIAB]) OR (niger[TIAB]) OR (rwanda[TIAB]) OR ("sierra leone"[TIAB]) OR ("south sudan"[TIAB]) OR (sudan[TIAB]) OR ("syrian arab republic"[TIAB]) OR (syria[TIAB]) OR (tajikistan[TIAB]) OR (togo[TIAB]) OR (uganda[TIAB]) OR (yemen[TIAB]) OR (angola[TIAB]) OR (algeria[TIAB]) OR (bangladesh[TIAB]) OR (benin[TIAB]) OR (bhutan[TIAB]) OR (bolivia[TIAB]) OR ("cabo verde"[TIAB]) OR (cambodia[TIAB]) OR (cameroon[TIAB]) OR (comoros[TIAB]) OR ("cote d'ivoire"[TIAB]) OR ("Côte d'Ivoire"[TIAB]) OR (Djibouti[TIAB]) OR (Egypt[TIAB]) OR ("El Salvador"[TIAB]) OR (Eswatini[TIAB]) OR (Ghana[TIAB]) OR (Honduras[TIAB]) OR (India[TIAB]) OR (kenya[TIAB]) OR (Kiribati[TIAB]) OR ("Kyrgyz Republic"[TIAB]) OR ("Lao PDR"[TIAB]) OR (Laos[TIAB]) OR (Lesotho[TIAB]) OR (Mauritania[TIAB]) OR (Micronesia[TIAB]) OR (Moldova[TIAB]) OR (Mongolia[TIAB]) OR (Morocco[TIAB]) OR (Myanmar[TIAB]) OR (Nepal[TIAB]) OR (Nicaragua[TIAB]) OR (Nigeria[TIAB]) OR (Pakistan[TIAB]) OR ("Papua New Guinea"[TIAB]) OR (Philippines[TIAB]) OR ("São Tomé and Principe"[TIAB]) OR (Senegal[TIAB]) OR ("Solomon Islands"[TIAB]) OR (SriLanka[TIAB]) OR ("Sri Lanka"[TIAB]) OR (Tanzania[TIAB]) OR ("Timor-Leste"[TIAB]) OR (Tunisia[TIAB]) OR (Ukraine[TIAB]) OR (Uzbekistan[TIAB]) OR (Vanuatu[TIAB]) OR (Vietnam[TIAB]) OR ("West Bank"[TIAB]) OR (Gaza[TIAB]) OR (Zambia[TIAB]) OR (Zimbabwe[TIAB]) OR (Albania[TIAB]) OR ("American Samoa"[TIAB]) OR (Argentina[TIAB]) OR (Armenia[TIAB]) OR (Azerbaijan[TIAB]) OR (Belarus[TIAB]) OR (Belize[TIAB]) OR (Bosnia[TIAB]) OR (Herzegovina[TIAB]) OR (Botswana[TIAB]) OR (Brazil[TIAB]) OR (Bulgaria[TIAB]) OR (China[TIAB]) OR (Colombia[TIAB]) OR ("Costa Rica"[TIAB]) OR (Cuba[TIAB]) OR (dominica[TIAB]) OR ("dominican republic"[TIAB]) OR ("equatorial guinea"[TIAB]) OR (ecuador[TIAB]) OR (fiji[TIAB]) OR (gabon[TIAB]) OR (georgia[TIAB]) OR (grenada[TIAB]) OR (guatemala[TIAB]) OR (guyana[TIAB]) OR (indonesia[TIAB]) OR (iran[TIAB]) OR (iraq[TIAB]) OR (jamaica[TIAB]) OR (jordan[TIAB]) OR (Kazakhstan[TIAB]) OR (kosovo[TIAB]) OR (lebanon[TIAB]) OR (libya[TIAB]) OR (malaysia[TIAB]) OR (maldives[TIAB]) OR ("marshall islands"[TIAB]) OR (mexico[TIAB]) OR (montenegro[TIAB]) OR (namibia[TIAB]) OR ("north macedonia"[TIAB]) OR (paraguay[TIAB]) OR (peru[TIAB]) OR ("russian federation"[TIAB]) OR (russia[TIAB]) OR (samoa[TIAB]) OR (serbia[TIAB]) OR ("south africa"[TIAB]) OR ("st. lucia"[TIAB]) OR ("st. vincent"[TIAB]) OR (grenadines[TIAB]) OR (suriname[TIAB]) OR (thailand[TIAB]) OR (tonga[TIAB]) OR (turkey[TIAB]) OR (Turkmenistan[TIAB]) OR (tuvalu[TIAB]) OR (venezuela[TIAB]) OR (asia[TIAB]) OR (pacific[TIAB]) OR ("latin america"[TIAB]) OR (Caribbean[TIAB]) OR ("middle east"[TIAB]) OR (Africa[TIAB]) OR ("North africa"[TIAB]) OR ("east asia"[TIAB]) OR ("south asia"[TIAB]) OR ("sub-Saharan Africa"[TIAB]) OR ("east africa"[TIAB]) OR ("west africa"[TIAB]) OR ("north africa"[TIAB]) OR ("low-income"[TIAB]) OR ("lower-middle-income"[TIAB]) OR ("upper-middle-income"[TIAB]) OR ("low middle-income"[TIAB])) AND ((HIV infection[MeSH Terms]) OR (acquired immune deficiency syndrome[MeSH Terms]) OR (HIV[TIAB]) OR ("AIDS"[TIAB]) OR ("acquired immune deficiency syndrome"[TIAB]) OR ("acquired immunodeficiency syndrome"[TIAB])) AND (2000/1/1:2021/4/1[pdat]) AND ((prevention[TIAB]) OR (prevent[TIAB])) |
| Diagnosis/Linkage | (((((((HIV testing[MeSH Terms]) OR (HIV prevent*[TIAB])) OR (link*[TIAB])) OR (self-test*[TIAB]) AND (2000/1/1:2021/4/1[pdat])) AND ((HIV infection[MeSH Terms]) OR (acquired immune deficiency syndrome[MeSH Terms]) OR (HIV[TIAB]) OR ("AIDS"[TIAB]) OR ("acquired immune deficiency syndrome"[TIAB]) OR ("acquired immunodeficiency syndrome"[TIAB]) AND (2000/1/1:2021/4/1[pdat]))) AND ((Afghanistan[TIAB]) OR ("Burkina Faso"[TIAB]) OR (Burundi[TIAB]) OR ("Central African Republic"[TIAB]) OR (Chad[TIAB]) OR (congo[TIAB]) OR (Eritrea[TIAB]) OR (Ethiopia[TIAB]) OR (gambia[TIAB]) OR (guinea[TIAB]) OR ("Guinea-Bissau"[TIAB]) OR (Haiti[TIAB]) OR ("Democratic People's Republic of Korea"[Mesh]) OR (korea[TIAB]) OR (liberia[TIAB]) OR (madagascar[TIAB]) OR (malawi[TIAB]) OR (mali[TIAB]) OR (mozambique[TIAB]) OR (niger[TIAB]) OR (rwanda[TIAB]) OR ("sierra leone"[TIAB]) OR ("south sudan"[TIAB]) OR (sudan[TIAB]) OR ("syrian arab republic"[TIAB]) OR (syria[TIAB]) OR (tajikistan[TIAB]) OR (togo[TIAB]) OR (uganda[TIAB]) OR (yemen[TIAB]) OR (angola[TIAB]) OR (algeria[TIAB]) OR (bangladesh[TIAB]) OR (benin[TIAB]) OR (bhutan[TIAB]) OR (bolivia[TIAB]) OR ("cabo verde"[TIAB]) OR (cambodia[TIAB]) OR (cameroon[TIAB]) OR (comoros[TIAB]) OR ("cote d'ivoire"[TIAB]) OR ("Côte d'Ivoire"[TIAB]) OR (Djibouti[TIAB]) OR (Egypt[TIAB]) OR ("El Salvador"[TIAB]) OR (Eswatini[TIAB]) OR (Ghana[TIAB]) OR (Honduras[TIAB]) OR (India[TIAB]) OR (kenya[TIAB]) OR (Kiribati[TIAB]) OR ("Kyrgyz Republic"[TIAB]) OR ("Lao PDR"[TIAB]) OR (Laos[TIAB]) OR (Lesotho[TIAB]) OR (Mauritania[TIAB]) OR (Micronesia[TIAB]) OR (Moldova[TIAB]) OR (Mongolia[TIAB]) OR (Morocco[TIAB]) OR (Myanmar[TIAB]) OR (Nepal[TIAB]) OR (Nicaragua[TIAB]) OR (Nigeria[TIAB]) OR (Pakistan[TIAB]) OR ("Papua New Guinea"[TIAB]) OR (Philippines[TIAB]) OR ("São Tomé and Principe"[TIAB]) OR (Senegal[TIAB]) OR ("Solomon Islands"[TIAB]) OR (SriLanka[TIAB]) OR ("Sri Lanka"[TIAB]) OR (Tanzania[TIAB]) OR ("Timor-Leste"[TIAB]) OR (Tunisia[TIAB]) OR (Ukraine[TIAB]) OR (Uzbekistan[TIAB]) OR (Vanuatu[TIAB]) OR (Vietnam[TIAB]) OR ("West Bank"[TIAB]) OR (Gaza[TIAB]) OR (Zambia[TIAB]) OR (Zimbabwe[TIAB]) OR (Albania[TIAB]) OR ("American Samoa"[TIAB]) OR (Argentina[TIAB]) OR (Armenia[TIAB]) OR (Azerbaijan[TIAB]) OR (Belarus[TIAB]) OR (Belize[TIAB]) OR (Bosnia[TIAB]) OR (Herzegovina[TIAB]) OR (Botswana[TIAB]) OR (Brazil[TIAB]) OR (Bulgaria[TIAB]) OR (China[TIAB]) OR (Colombia[TIAB]) OR ("Costa Rica"[TIAB]) OR (Cuba[TIAB]) OR (dominica[TIAB]) OR ("dominican republic"[TIAB]) OR ("equatorial guinea"[TIAB]) OR (ecuador[TIAB]) OR (fiji[TIAB]) OR (gabon[TIAB]) OR (georgia[TIAB]) OR (grenada[TIAB]) OR (guatemala[TIAB]) OR (guyana[TIAB]) OR (indonesia[TIAB]) OR (iran[TIAB]) OR (iraq[TIAB]) OR (jamaica[TIAB]) OR (jordan[TIAB]) OR (Kazakhstan[TIAB]) OR (kosovo[TIAB]) OR (lebanon[TIAB]) OR (libya[TIAB]) OR (malaysia[TIAB]) OR (maldives[TIAB]) OR ("marshall islands"[TIAB]) OR (mexico[TIAB]) OR (montenegro[TIAB]) OR (namibia[TIAB]) OR ("north macedonia"[TIAB]) OR (paraguay[TIAB]) OR (peru[TIAB]) OR ("russian federation"[TIAB]) OR (russia[TIAB]) OR (samoa[TIAB]) OR (serbia[TIAB]) OR ("south africa"[TIAB]) OR ("st. lucia"[TIAB]) OR ("st. vincent"[TIAB]) OR (grenadines[TIAB]) OR (suriname[TIAB]) OR (thailand[TIAB]) OR (tonga[TIAB]) OR (turkey[TIAB]) OR (Turkmenistan[TIAB]) OR (tuvalu[TIAB]) OR (venezuela[TIAB]) OR (asia[TIAB]) OR (pacific[TIAB]) OR ("latin america"[TIAB]) OR (Caribbean[TIAB]) OR ("middle east"[TIAB]) OR (Africa[TIAB]) OR ("North africa"[TIAB]) OR ("east asia"[TIAB]) OR ("south asia"[TIAB]) OR ("sub-Saharan Africa"[TIAB]) OR ("east africa"[TIAB]) OR ("west africa"[TIAB]) OR ("north africa"[TIAB]) OR ("low-income"[TIAB]) OR ("lower-middle-income"[TIAB]) OR ("upper-middle-income"[TIAB]) OR ("low middle-income"[TIAB]) AND (2000/1/1:2021/4/1[pdat]))) AND ((cell phone[MeSH Terms]) OR (smartphone[MeSH Terms]) OR (computer, handheld[MeSH Terms]) OR (wireless technology[MeSH Terms]) OR (internet[MeSH Terms]) OR (mhealth[TIAB]) OR (social media[MeSH Terms]) OR (mobile applications[MeSH Terms]) OR (app[TIAB]) OR (web[TIAB]) OR (digital*[TIAB]) OR (internet[TIAB]) OR ("sms"[TIAB]) OR (whatsapp[TIAB]) OR (facebook[TIAB]) OR (grindr[TIAB]) OR (telegram[TIAB]) OR (tiktok[TIAB]) OR (geosocial[TIAB]) OR (mxit[TIAB]) AND (2000/1/1:2021/4/1[pdat]))) AND ((adolescent[MeSH Terms]) OR (young adult[MeSH Terms]) OR (teen*) OR (youth*) OR (adoles*) OR (young adult*) AND (2000/1/1:2021/4/1[pdat])) |
| ART Adherence/Retention | ((adolescent[MeSH Terms]) OR (young adult[MeSH Terms]) OR (teen*) OR (youth*) OR (adoles*) OR (young adult*)) AND ((cell phone[MeSH Terms]) OR (smartphone[MeSH Terms]) OR (computer, handheld[MeSH Terms]) OR (wireless technology[MeSH Terms]) OR (internet[MeSH Terms]) OR (mhealth[TIAB]) OR (social media[MeSH Terms]) OR (mobile applications[MeSH Terms]) OR (app[TIAB]) OR (web[TIAB]) OR (digital*[TIAB]) OR (internet[TIAB]) OR ("sms"[TIAB]) OR (whatsapp[TIAB]) OR (facebook[TIAB]) OR (grindr[TIAB]) OR (telegram[TIAB]) OR (tiktok[TIAB]) OR (geosocial[TIAB]) OR (mxit[TIAB])) AND ((Afghanistan[TIAB]) OR ("Burkina Faso"[TIAB]) OR (Burundi[TIAB]) OR ("Central African Republic"[TIAB]) OR (Chad[TIAB]) OR (congo[TIAB]) OR (Eritrea[TIAB]) OR (Ethiopia[TIAB]) OR (gambia[TIAB]) OR (guinea[TIAB]) OR ("Guinea-Bissau"[TIAB]) OR (Haiti[TIAB]) OR ("Democratic People's Republic of Korea"[Mesh]) OR (korea[TIAB]) OR (liberia[TIAB]) OR (madagascar[TIAB]) OR (malawi[TIAB]) OR (mali[TIAB]) OR (mozambique[TIAB]) OR (niger[TIAB]) OR (rwanda[TIAB]) OR ("sierra leone"[TIAB]) OR ("south sudan"[TIAB]) OR (sudan[TIAB]) OR ("syrian arab republic"[TIAB]) OR (syria[TIAB]) OR (tajikistan[TIAB]) OR (togo[TIAB]) OR (uganda[TIAB]) OR (yemen[TIAB]) OR (angola[TIAB]) OR (algeria[TIAB]) OR (bangladesh[TIAB]) OR (benin[TIAB]) OR (bhutan[TIAB]) OR (bolivia[TIAB]) OR ("cabo verde"[TIAB]) OR (cambodia[TIAB]) OR (cameroon[TIAB]) OR (comoros[TIAB]) OR ("cote d'ivoire"[TIAB]) OR ("Côte d'Ivoire"[TIAB]) OR (Djibouti[TIAB]) OR (Egypt[TIAB]) OR ("El Salvador"[TIAB]) OR (Eswatini[TIAB]) OR (Ghana[TIAB]) OR (Honduras[TIAB]) OR (India[TIAB]) OR (kenya[TIAB]) OR (Kiribati[TIAB]) OR ("Kyrgyz Republic"[TIAB]) OR ("Lao PDR"[TIAB]) OR (Laos[TIAB]) OR (Lesotho[TIAB]) OR (Mauritania[TIAB]) OR (Micronesia[TIAB]) OR (Moldova[TIAB]) OR (Mongolia[TIAB]) OR (Morocco[TIAB]) OR (Myanmar[TIAB]) OR (Nepal[TIAB]) OR (Nicaragua[TIAB]) OR (Nigeria[TIAB]) OR (Pakistan[TIAB]) OR ("Papua New Guinea"[TIAB]) OR (Philippines[TIAB]) OR ("São Tomé and Principe"[TIAB]) OR (Senegal[TIAB]) OR ("Solomon Islands"[TIAB]) OR (SriLanka[TIAB]) OR ("Sri Lanka"[TIAB]) OR (Tanzania[TIAB]) OR ("Timor-Leste"[TIAB]) OR (Tunisia[TIAB]) OR (Ukraine[TIAB]) OR (Uzbekistan[TIAB]) OR (Vanuatu[TIAB]) OR (Vietnam[TIAB]) OR ("West Bank"[TIAB]) OR (Gaza[TIAB]) OR (Zambia[TIAB]) OR (Zimbabwe[TIAB]) OR (Albania[TIAB]) OR ("American Samoa"[TIAB]) OR (Argentina[TIAB]) OR (Armenia[TIAB]) OR (Azerbaijan[TIAB]) OR (Belarus[TIAB]) OR (Belize[TIAB]) OR (Bosnia[TIAB]) OR (Herzegovina[TIAB]) OR (Botswana[TIAB]) OR (Brazil[TIAB]) OR (Bulgaria[TIAB]) OR (China[TIAB]) OR (Colombia[TIAB]) OR ("Costa Rica"[TIAB]) OR (Cuba[TIAB]) OR (dominica[TIAB]) OR ("dominican republic"[TIAB]) OR ("equatorial guinea"[TIAB]) OR (ecuador[TIAB]) OR (fiji[TIAB]) OR (gabon[TIAB]) OR (georgia[TIAB]) OR (grenada[TIAB]) OR (guatemala[TIAB]) OR (guyana[TIAB]) OR (indonesia[TIAB]) OR (iran[TIAB]) OR (iraq[TIAB]) OR (jamaica[TIAB]) OR (jordan[TIAB]) OR (Kazakhstan[TIAB]) OR (kosovo[TIAB]) OR (lebanon[TIAB]) OR (libya[TIAB]) OR (malaysia[TIAB]) OR (maldives[TIAB]) OR ("marshall islands"[TIAB]) OR (mexico[TIAB]) OR (montenegro[TIAB]) OR (namibia[TIAB]) OR ("north macedonia"[TIAB]) OR (paraguay[TIAB]) OR (peru[TIAB]) OR ("russian federation"[TIAB]) OR (russia[TIAB]) OR (samoa[TIAB]) OR (serbia[TIAB]) OR ("south africa"[TIAB]) OR ("st. lucia"[TIAB]) OR ("st. vincent"[TIAB]) OR (grenadines[TIAB]) OR (suriname[TIAB]) OR (thailand[TIAB]) OR (tonga[TIAB]) OR (turkey[TIAB]) OR (Turkmenistan[TIAB]) OR (tuvalu[TIAB]) OR (venezuela[TIAB]) OR (asia[TIAB]) OR (pacific[TIAB]) OR ("latin america"[TIAB]) OR (Caribbean[TIAB]) OR ("middle east"[TIAB]) OR (Africa[TIAB]) OR ("North africa"[TIAB]) OR ("east asia"[TIAB]) OR ("south asia"[TIAB]) OR ("sub-Saharan Africa"[TIAB]) OR ("east africa"[TIAB]) OR ("west africa"[TIAB]) OR ("north africa"[TIAB]) OR ("low-income"[TIAB]) OR ("lower-middle-income"[TIAB]) OR ("upper-middle-income"[TIAB]) OR ("low middle-income"[TIAB])) AND ((HIV infection[MeSH Terms]) OR (acquired immune deficiency syndrome[MeSH Terms]) OR (HIV[TIAB]) OR ("AIDS"[TIAB]) OR ("acquired immune deficiency syndrome"[TIAB]) OR ("acquired immunodeficiency syndrome"[TIAB])) AND (2000/1/1:2021/4/1[pdat]) AND ((adheren*[TIAB]) OR (retention[TIAB]) OR ("retention in care"[TIAB]) OR ("Treatment Adherence and Compliance"[Mesh]) OR ("Retention in Care"[Mesh])) |
| Viral Suppression | ((adolescent[MeSH Terms]) OR (young adult[MeSH Terms]) OR (teen*) OR (youth*) OR (adoles*) OR (young adult*)) AND ((cell phone[MeSH Terms]) OR (smartphone[MeSH Terms]) OR (computer, handheld[MeSH Terms]) OR (wireless technology[MeSH Terms]) OR (internet[MeSH Terms]) OR (mhealth[TIAB]) OR (social media[MeSH Terms]) OR (mobile applications[MeSH Terms]) OR (app[TIAB]) OR (web[TIAB]) OR (digital*[TIAB]) OR (internet[TIAB]) OR ("sms"[TIAB]) OR (whatsapp[TIAB]) OR (facebook[TIAB]) OR (grindr[TIAB]) OR (telegram[TIAB]) OR (tiktok[TIAB]) OR (geosocial[TIAB]) OR (mxit[TIAB])) AND ((Afghanistan[TIAB]) OR ("Burkina Faso"[TIAB]) OR (Burundi[TIAB]) OR ("Central African Republic"[TIAB]) OR (Chad[TIAB]) OR (congo[TIAB]) OR (Eritrea[TIAB]) OR (Ethiopia[TIAB]) OR (gambia[TIAB]) OR (guinea[TIAB]) OR ("Guinea-Bissau"[TIAB]) OR (Haiti[TIAB]) OR ("Democratic People's Republic of Korea"[Mesh]) OR (korea[TIAB]) OR (liberia[TIAB]) OR (madagascar[TIAB]) OR (malawi[TIAB]) OR (mali[TIAB]) OR (mozambique[TIAB]) OR (niger[TIAB]) OR (rwanda[TIAB]) OR ("sierra leone"[TIAB]) OR ("south sudan"[TIAB]) OR (sudan[TIAB]) OR ("syrian arab republic"[TIAB]) OR (syria[TIAB]) OR (tajikistan[TIAB]) OR (togo[TIAB]) OR (uganda[TIAB]) OR (yemen[TIAB]) OR (angola[TIAB]) OR (algeria[TIAB]) OR (bangladesh[TIAB]) OR (benin[TIAB]) OR (bhutan[TIAB]) OR (bolivia[TIAB]) OR ("cabo verde"[TIAB]) OR (cambodia[TIAB]) OR (cameroon[TIAB]) OR (comoros[TIAB]) OR ("cote d'ivoire"[TIAB]) OR ("Côte d'Ivoire"[TIAB]) OR (Djibouti[TIAB]) OR (Egypt[TIAB]) OR ("El Salvador"[TIAB]) OR (Eswatini[TIAB]) OR (Ghana[TIAB]) OR (Honduras[TIAB]) OR (India[TIAB]) OR (kenya[TIAB]) OR (Kiribati[TIAB]) OR ("Kyrgyz Republic"[TIAB]) OR ("Lao PDR"[TIAB]) OR (Laos[TIAB]) OR (Lesotho[TIAB]) OR (Mauritania[TIAB]) OR (Micronesia[TIAB]) OR (Moldova[TIAB]) OR (Mongolia[TIAB]) OR (Morocco[TIAB]) OR (Myanmar[TIAB]) OR (Nepal[TIAB]) OR (Nicaragua[TIAB]) OR (Nigeria[TIAB]) OR (Pakistan[TIAB]) OR ("Papua New Guinea"[TIAB]) OR (Philippines[TIAB]) OR ("São Tomé and Principe"[TIAB]) OR (Senegal[TIAB]) OR ("Solomon Islands"[TIAB]) OR (SriLanka[TIAB]) OR ("Sri Lanka"[TIAB]) OR (Tanzania[TIAB]) OR ("Timor-Leste"[TIAB]) OR (Tunisia[TIAB]) OR (Ukraine[TIAB]) OR (Uzbekistan[TIAB]) OR (Vanuatu[TIAB]) OR (Vietnam[TIAB]) OR ("West Bank"[TIAB]) OR (Gaza[TIAB]) OR (Zambia[TIAB]) OR (Zimbabwe[TIAB]) OR (Albania[TIAB]) OR ("American Samoa"[TIAB]) OR (Argentina[TIAB]) OR (Armenia[TIAB]) OR (Azerbaijan[TIAB]) OR (Belarus[TIAB]) OR (Belize[TIAB]) OR (Bosnia[TIAB]) OR (Herzegovina[TIAB]) OR (Botswana[TIAB]) OR (Brazil[TIAB]) OR (Bulgaria[TIAB]) OR (China[TIAB]) OR (Colombia[TIAB]) OR ("Costa Rica"[TIAB]) OR (Cuba[TIAB]) OR (dominica[TIAB]) OR ("dominican republic"[TIAB]) OR ("equatorial guinea"[TIAB]) OR (ecuador[TIAB]) OR (fiji[TIAB]) OR (gabon[TIAB]) OR (georgia[TIAB]) OR (grenada[TIAB]) OR (guatemala[TIAB]) OR (guyana[TIAB]) OR (indonesia[TIAB]) OR (iran[TIAB]) OR (iraq[TIAB]) OR (jamaica[TIAB]) OR (jordan[TIAB]) OR (Kazakhstan[TIAB]) OR (kosovo[TIAB]) OR (lebanon[TIAB]) OR (libya[TIAB]) OR (malaysia[TIAB]) OR (maldives[TIAB]) OR ("marshall islands"[TIAB]) OR (mexico[TIAB]) OR (montenegro[TIAB]) OR (namibia[TIAB]) OR ("north macedonia"[TIAB]) OR (paraguay[TIAB]) OR (peru[TIAB]) OR ("russian federation"[TIAB]) OR (russia[TIAB]) OR (samoa[TIAB]) OR (serbia[TIAB]) OR ("south africa"[TIAB]) OR ("st. lucia"[TIAB]) OR ("st. vincent"[TIAB]) OR (grenadines[TIAB]) OR (suriname[TIAB]) OR (thailand[TIAB]) OR (tonga[TIAB]) OR (turkey[TIAB]) OR (Turkmenistan[TIAB]) OR (tuvalu[TIAB]) OR (venezuela[TIAB]) OR (asia[TIAB]) OR (pacific[TIAB]) OR ("latin america"[TIAB]) OR (Caribbean[TIAB]) OR ("middle east"[TIAB]) OR (Africa[TIAB]) OR ("North africa"[TIAB]) OR ("east asia"[TIAB]) OR ("south asia"[TIAB]) OR ("sub-Saharan Africa"[TIAB]) OR ("east africa"[TIAB]) OR ("west africa"[TIAB]) OR ("north africa"[TIAB]) OR ("low-income"[TIAB]) OR ("lower-middle-income"[TIAB]) OR ("upper-middle-income"[TIAB]) OR ("low middle-income"[TIAB])) AND ((HIV infection[MeSH Terms]) OR (acquired immune deficiency syndrome[MeSH Terms]) OR (HIV[TIAB]) OR ("AIDS"[TIAB]) OR ("acquired immune deficiency syndrome"[TIAB]) OR ("acquired immunodeficiency syndrome"[TIAB])) AND (2000/1/1:2021/4/1[pdat]) AND ((viral suppression[TIAB]) OR (viral load[TIAB])) |
| Transition to Adult Care | ((adolescent[MeSH Terms]) OR (young adult[MeSH Terms]) OR (teen*) OR (youth*) OR (adoles*) OR (young adult*)) AND ((cell phone[MeSH Terms]) OR (smartphone[MeSH Terms]) OR (computer, handheld[MeSH Terms]) OR (wireless technology[MeSH Terms]) OR (internet[MeSH Terms]) OR (mhealth[TIAB]) OR (social media[MeSH Terms]) OR (mobile applications[MeSH Terms]) OR (app[TIAB]) OR (web[TIAB]) OR (digital*[TIAB]) OR (internet[TIAB]) OR ("sms"[TIAB]) OR (whatsapp[TIAB]) OR (facebook[TIAB]) OR (grindr[TIAB]) OR (telegram[TIAB]) OR (tiktok[TIAB]) OR (geosocial[TIAB]) OR (mxit[TIAB])) AND ((Afghanistan[TIAB]) OR ("Burkina Faso"[TIAB]) OR (Burundi[TIAB]) OR ("Central African Republic"[TIAB]) OR (Chad[TIAB]) OR (congo[TIAB]) OR (Eritrea[TIAB]) OR (Ethiopia[TIAB]) OR (gambia[TIAB]) OR (guinea[TIAB]) OR ("Guinea-Bissau"[TIAB]) OR (Haiti[TIAB]) OR ("Democratic People's Republic of Korea"[Mesh]) OR (korea[TIAB]) OR (liberia[TIAB]) OR (madagascar[TIAB]) OR (malawi[TIAB]) OR (mali[TIAB]) OR (mozambique[TIAB]) OR (niger[TIAB]) OR (rwanda[TIAB]) OR ("sierra leone"[TIAB]) OR ("south sudan"[TIAB]) OR (sudan[TIAB]) OR ("syrian arab republic"[TIAB]) OR (syria[TIAB]) OR (tajikistan[TIAB]) OR (togo[TIAB]) OR (uganda[TIAB]) OR (yemen[TIAB]) OR (angola[TIAB]) OR (algeria[TIAB]) OR (bangladesh[TIAB]) OR (benin[TIAB]) OR (bhutan[TIAB]) OR (bolivia[TIAB]) OR ("cabo verde"[TIAB]) OR (cambodia[TIAB]) OR (cameroon[TIAB]) OR (comoros[TIAB]) OR ("cote d'ivoire"[TIAB]) OR ("Côte d'Ivoire"[TIAB]) OR (Djibouti[TIAB]) OR (Egypt[TIAB]) OR ("El Salvador"[TIAB]) OR (Eswatini[TIAB]) OR (Ghana[TIAB]) OR (Honduras[TIAB]) OR (India[TIAB]) OR (kenya[TIAB]) OR (Kiribati[TIAB]) OR ("Kyrgyz Republic"[TIAB]) OR ("Lao PDR"[TIAB]) OR (Laos[TIAB]) OR (Lesotho[TIAB]) OR (Mauritania[TIAB]) OR (Micronesia[TIAB]) OR (Moldova[TIAB]) OR (Mongolia[TIAB]) OR (Morocco[TIAB]) OR (Myanmar[TIAB]) OR (Nepal[TIAB]) OR (Nicaragua[TIAB]) OR (Nigeria[TIAB]) OR (Pakistan[TIAB]) OR ("Papua New Guinea"[TIAB]) OR (Philippines[TIAB]) OR ("São Tomé and Principe"[TIAB]) OR (Senegal[TIAB]) OR ("Solomon Islands"[TIAB]) OR (SriLanka[TIAB]) OR ("Sri Lanka"[TIAB]) OR (Tanzania[TIAB]) OR ("Timor-Leste"[TIAB]) OR (Tunisia[TIAB]) OR (Ukraine[TIAB]) OR (Uzbekistan[TIAB]) OR (Vanuatu[TIAB]) OR (Vietnam[TIAB]) OR ("West Bank"[TIAB]) OR (Gaza[TIAB]) OR (Zambia[TIAB]) OR (Zimbabwe[TIAB]) OR (Albania[TIAB]) OR ("American Samoa"[TIAB]) OR (Argentina[TIAB]) OR (Armenia[TIAB]) OR (Azerbaijan[TIAB]) OR (Belarus[TIAB]) OR (Belize[TIAB]) OR (Bosnia[TIAB]) OR (Herzegovina[TIAB]) OR (Botswana[TIAB]) OR (Brazil[TIAB]) OR (Bulgaria[TIAB]) OR (China[TIAB]) OR (Colombia[TIAB]) OR ("Costa Rica"[TIAB]) OR (Cuba[TIAB]) OR (dominica[TIAB]) OR ("dominican republic"[TIAB]) OR ("equatorial guinea"[TIAB]) OR (ecuador[TIAB]) OR (fiji[TIAB]) OR (gabon[TIAB]) OR (georgia[TIAB]) OR (grenada[TIAB]) OR (guatemala[TIAB]) OR (guyana[TIAB]) OR (indonesia[TIAB]) OR (iran[TIAB]) OR (iraq[TIAB]) OR (jamaica[TIAB]) OR (jordan[TIAB]) OR (Kazakhstan[TIAB]) OR (kosovo[TIAB]) OR (lebanon[TIAB]) OR (libya[TIAB]) OR (malaysia[TIAB]) OR (maldives[TIAB]) OR ("marshall islands"[TIAB]) OR (mexico[TIAB]) OR (montenegro[TIAB]) OR (namibia[TIAB]) OR ("north macedonia"[TIAB]) OR (paraguay[TIAB]) OR (peru[TIAB]) OR ("russian federation"[TIAB]) OR (russia[TIAB]) OR (samoa[TIAB]) OR (serbia[TIAB]) OR ("south africa"[TIAB]) OR ("st. lucia"[TIAB]) OR ("st. vincent"[TIAB]) OR (grenadines[TIAB]) OR (suriname[TIAB]) OR (thailand[TIAB]) OR (tonga[TIAB]) OR (turkey[TIAB]) OR (Turkmenistan[TIAB]) OR (tuvalu[TIAB]) OR (venezuela[TIAB]) OR (asia[TIAB]) OR (pacific[TIAB]) OR ("latin america"[TIAB]) OR (Caribbean[TIAB]) OR ("middle east"[TIAB]) OR (Africa[TIAB]) OR ("North africa"[TIAB]) OR ("east asia"[TIAB]) OR ("south asia"[TIAB]) OR ("sub-Saharan Africa"[TIAB]) OR ("east africa"[TIAB]) OR ("west africa"[TIAB]) OR ("north africa"[TIAB]) OR ("low-income"[TIAB]) OR ("lower-middle-income"[TIAB]) OR ("upper-middle-income"[TIAB]) OR ("low middle-income"[TIAB])) AND ((HIV infection[MeSH Terms]) OR (acquired immune deficiency syndrome[MeSH Terms]) OR (HIV[TIAB]) OR ("AIDS"[TIAB]) OR ("acquired immune deficiency syndrome"[TIAB]) OR ("acquired immunodeficiency syndrome"[TIAB])) AND (2000/1/1:2021/4/1[pdat]) AND ((transition[TIAB]) OR ("healthcare transition"[TIAB]) OR ("pediatric to adult care"[Mesh]) OR ("health care transition"[Mesh])) |
